# Impact of Polymicrobial Infection on Fitness of Streptococcus gordonii *In Vivo*

**DOI:** 10.1128/mbio.00658-23

**Published:** 2023-04-12

**Authors:** Satya D. Pandey, John D. Perpich, Kendall S. Stocke, Jillian M. Mansfield, Yuichiro Kikuchi, Lan Yakoumatos, Artur Muszyński, Parastoo Azadi, Hervé Tettelin, Marvin Whiteley, Silvia M. Uriarte, Juhi Bagaitkar, Margaret Vickerman, Richard J. Lamont

**Affiliations:** a Department of Oral Immunology and Infectious Diseases, University of Louisville, Louisville, Kentucky, USA; b Department of Pharmaceutical Science, Sullivan University, Louisville, Kentucky, USA; c Department of Oral Biology, School of Dental Medicine, University at Buffalo, Buffalo, New York, USA; d Department of Biochemistry and Molecular Biology, University of Georgia, Athens, Georgia, USA; e Department of Microbiology and Immunology, Institute for Genome Sciences, University of Maryland School of Medicine, Baltimore, Maryland, USA; f School of Biological Sciences, Center for Microbial Dynamics and Infection, Georgia Institute of Technology, Atlanta, Georgia, USA; g Center for Microbial Pathogenesis, Nationwide Children's Hospital, Columbus, Ohio, USA; h Department of Pediatrics, The Ohio State College of Medicine, Columbus, Ohio, USA; The University of Kansas Medical Center

**Keywords:** neutrophil killing, polymicrobial synergy, receptor polysaccharide

## Abstract

Pathogenic microbial ecosystems are often polymicrobial, and interbacterial interactions drive emergent properties of these communities. In the oral cavity, Streptococcus gordonii is a foundational species in the development of plaque biofilms, which can contribute to periodontal disease and, after gaining access to the bloodstream, target remote sites such as heart valves. Here, we used a transposon sequencing (Tn-Seq) library of S. gordonii to identify genes that influence fitness in a murine abscess model, both as a monoinfection and as a coinfection with an oral partner species, Porphyromonas gingivalis. In the context of a monoinfection, conditionally essential genes were widely distributed among functional pathways. Coinfection with P. gingivalis almost completely changed the nature of *in vivo* gene essentiality. Community-dependent essential (CoDE) genes under the coinfection condition were primarily related to DNA replication, transcription, and translation, indicating that robust growth and replication are required to survive with P. gingivalis *in vivo*. Interestingly, a group of genes in an operon encoding streptococcal receptor polysaccharide (RPS) were associated with decreased fitness of S. gordonii in a coinfection with P. gingivalis. Individual deletion of two of these genes (SGO_2020 and SGO_2024) resulted in the loss of RPS production by S. gordonii and increased susceptibility to killing by neutrophils. P. gingivalis protected the RPS mutants by inhibiting neutrophil recruitment, degranulation, and neutrophil extracellular trap (NET) formation. These results provide insight into genes and functions that are important for S. gordonii survival *in vivo* and the nature of polymicrobial synergy with P. gingivalis. Furthermore, we show that RPS-mediated immune protection in S. gordonii is dispensable and detrimental in the presence of a synergistic partner species that can interfere with neutrophil killing mechanisms.

## INTRODUCTION

Many human microbial diseases, particularly those originating at mucosal barriers, involve polymicrobial assemblages of organisms that function as the etiological unit (1, 2). Bacteria within these communities are physically and metabolically integrated through coadhesion and the exchange of diffusible signaling molecules and metabolites (3–6). Such interspecies interactions can either enhance or suppress pathogenicity, depending on context, and examples of both outcomes are well documented (3, 7–12). The totality of intracommunity interactions and host-community interactions thus defines the course of the disease. In the oral cavity, periodontitis is a microbial community-driven disease in which a prolonged or uncontrolled inflammatory response contributes to tissue damage. Porphyromonas gingivalis is a major pathogen in periodontitis; however, the pathogenic state of P. gingivalis is dependent on the community context (13, 14). Synergistic pathogenicity has been shown to occur between P. gingivalis and a number of other oral organisms, including Fusobacterium nucleatum, Tannerella forsythia, Treponema denticola, and Streptococcus gordonii (15–18).

Mitis group streptococci such as S. gordonii are abundant early colonizers of newly erupted or cleaned tooth surfaces and are a major component of developing supragingival and subgingival biofilms (19). S. gordonii can provide an attachment substratum for partner species through receptor polysaccharide (RPS) and surface protein adhesins (20, 21), and can act as an accessory pathogen for P. gingivalis to increase community pathogenicity, or nososymbiocity, in periodontal disease (15, 22). However, this composite outcome belies a more nuanced and multidimensional relationship between the two organisms. S. gordonii secretes *para*-amino benzoic acid (pABA), which can be acquired by P. gingivalis and interferes with tyrosine phosphorylation/dephosphorylation-based signaling, resulting in lower pathogenicity in animal models (13, 23, 24). Physical association between the organisms, however, can both reduce the expression of pABA synthesis genes in S. gordonii and independently stimulate phosphotyrosine signaling in P. gingivalis, and thus physically integrated P. gingivalis and S. gordonii are ultimately more pathogenic in the context of periodontal tissue destruction. In contrast, at epithelial surfaces, peroxide produced by S. gordonii inactivates the P. gingivalis gingipain proteases and prevents proteolytic activation of the Notch signaling pathway in epithelial cells (25). In this context, S. gordonii functions as a homeostatic commensal.

In addition to the oral environment, oral bacteria, including S. gordonii and P. gingivalis, regularly gain access to the bloodstream following dental procedures or orodigestive functions such as mastication (26–28). The organisms can remain viable in blood and travel to and colonize remote organs. Colonization of damaged heart valves by S. gordonii can result in infective endocarditis (IE). P. gingivalis has an extensive impact on the expressed proteome of S. gordonii (29), and P. gingivalis may augment the survival of S. gordonii *in vivo*, in part by dampening the immune clearance of S. gordonii. For example, P. gingivalis can diminish macrophage killing of S. gordonii by modifying reactive oxygen species (ROS) production (30). This concept is also supported by reports that in a murine model, colonization by P. gingivalis increases the level of the commensal bacterial load, including streptococci (14).

While much is known regarding the outcome of interspecies interactions, less information is available regarding the genes that confer fitness in P. gingivalis or S. gordonii in coinfections. Our previous work using a transposon sequencing (Tn-Seq) library of P. gingivalis found that genes impacting fitness with S. gordonii included those involved in metabolism and energy production, along with cell wall and membrane biogenesis (31). The tyrosine kinase Ptk1 was also essential for coinfections with both S. gordonii and F. nucleatum (31). In the current study, we generated a Tn-Seq library in S. gordonii to identify genes which increase or decrease fitness in a coinfection with P. gingivalis. The operon encoding the RPS of S. gordonii was found to diminish fitness with P. gingivalis. In the absence of RPS, S. gordonii cells were more efficiently killed by neutrophils; however, P. gingivalis could compensate for this defect by inhibiting neutrophil functionality.

## RESULTS

### S. gordonii Tn-Seq *in vivo* screen.

**FIG 1:**
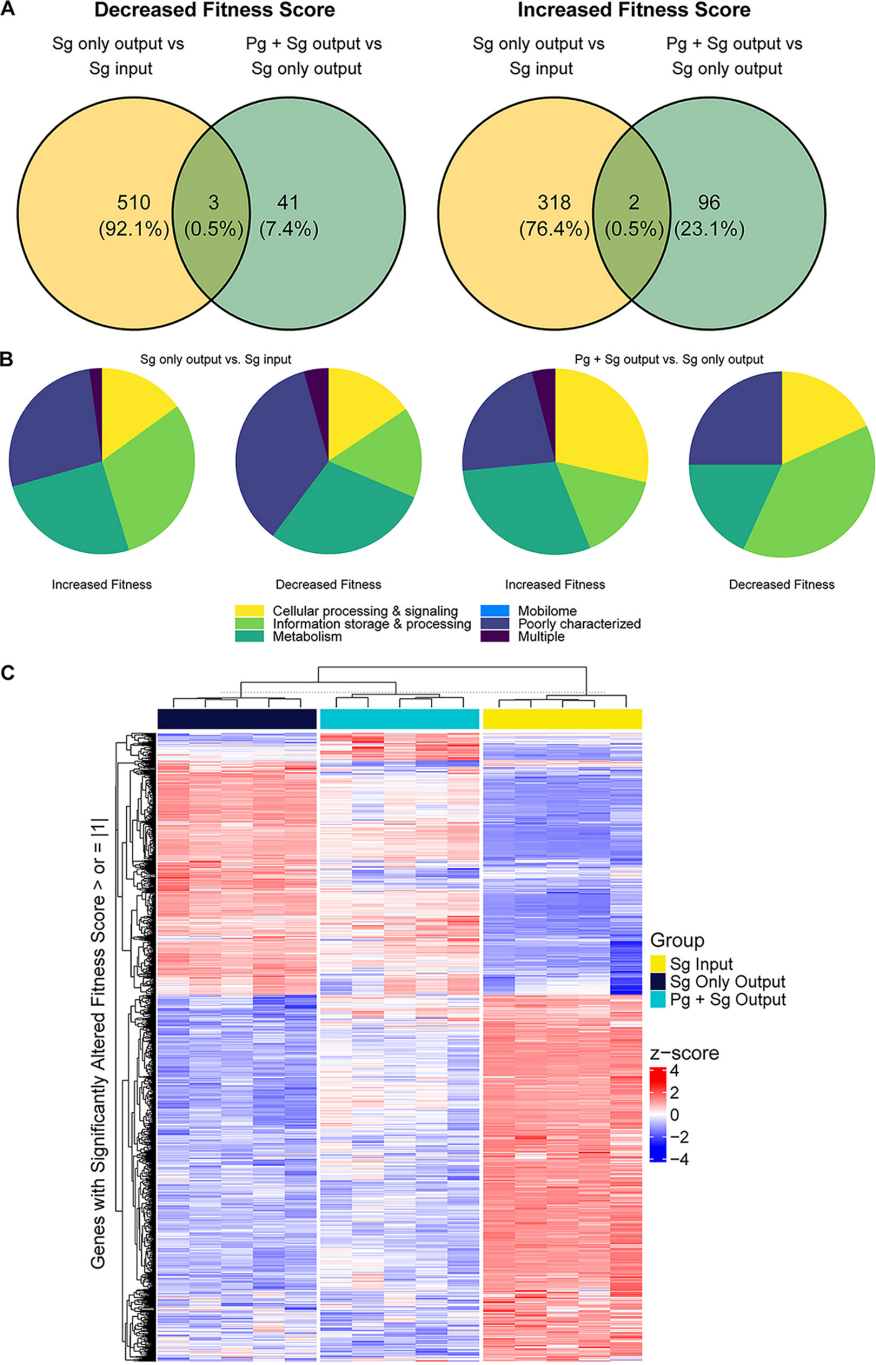
Summary of Tn-Seq data identifying S. gordonii (Sg) fitness determinants in infections with or without P. gingivalis (Pg). (A) Venn diagrams of genes showing negative or positive fitness scores in monoinfections and coinfections; (B) pie charts showing COG category distribution of *S. gordonii* genes showing a fitness effect in monoinfection or coinfection with *P. gingivalis*; (C) heat map showing Z-scores for genes with fitness effects in the monoinfection and coinfection groups.

We generated an S. gordonii Tn*5* transposon library to investigate genes required for fitness in an *in vivo* abscess model in the context of both a monoinfection and a dual-species infection with P. gingivalis. The Tn-Seq library consisted of ~37,300 colonies with a median of 282 unique barcodes/gene and reads with at least 5 fragments per kilobase per million (FPKM) in 1963 of 2,052 unique protein-coding genes. One hundred five genes were not represented in the input pool, or insertions were present at less than 1% of the expected value, and were thus considered essential under the *in vitro* growth conditions employed (see Table S1A in the supplemental material). We first looked at genes conditionally essential for S. gordonii growth in the abscess by comparing mutant abundance in the output pool of the S. gordonii-alone (Sg-only) group to the input pool. We found 513 genes in which transposon insertion decreased fitness, and these were broadly distributed across Clusters of Orthologous Groups (COGs) functional categories (Fig. 1A and B; Table S1B). Similarly, in the converse situation, examining mutants that were overrepresented in the output pool showed that there were 320 genes in which transposon insertion increased fitness, also broadly distributed across COG functional categories (Fig. 1A and B; Table S1C). Remarkably, coinfection with P. gingivalis (Sg+Pg group) almost completely rescued the fitness defect of all the genes essential for S. gordonii survival as a monoinfection, with only 3 genes in common between the two groups (Fig. 1A; Table S1D). Similarly, the presence of P. gingivalis resulted in major changes in genes that increased the fitness of S. gordonii when mutated, with only two genes in common (Fig. 1A; Table S1E). In both cases the coinfection condition revealed a smaller set of genes associated with decreased/increased fitness, with similar COG distribution patterns (Fig. 1A and B). The distinctiveness of the genes required for the fitness of S. gordonii alone compared to community-dependent essential (CoDE) genes (32, 33) is also illustrated by a heat map (Fig. 1C) and by volcano plots (see Fig. S1 in the supplemental material). Thus, the context of a dual-species community dramatically alters gene essentiality in S. gordonii.

10.1128/mbio.00658-23.1 FIG S1 Volcano plots showing mean fitness scores (log_10_ difference in abundance in the coinfection group relative to the monoinfection group) and *P* values (−log_10_) for S. gordonii (Sg) gene mutations in monoinfection (A) or coinfection with P. gingivalis (Pg) (B). Download FIG S1, PDF file, 0.3 MB. Copyright © 2023 Pandey et al. 2023 Pandey et al. https://creativecommons.org/licenses/by/4.0/ This content is distributed under the terms of the Creative Commons Attribution 4.0 International license.

10.1128/mbio.00658-23.9 TABLE S1 (A) All essential genes in *S. gordonii* input. (B) All genes demonstrating significantly decreased fitness after transposon insertion in *S. gordonii*-only (Sg-only) output versus *S. gordonii* input. (C) All genes demonstrating significantly increased fitness after transposon insertion in *S. gordonii*-only output versus *S. gordonii* input. (D) All genes demonstrating significantly decreased fitness after transposon insertion in *P. gingivalis* plus *S. gordonii* (Pg+Sg) output versus Sg-only output. (E) All genes demonstrating significantly increased fitness after transposon insertion in Pg+Sg output versus Sg-only output. Download Table S1, XLSX file, 0.1 MB. Copyright © 2023 Pandey et al. 2023 Pandey et al. https://creativecommons.org/licenses/by/4.0/ This content is distributed under the terms of the Creative Commons Attribution 4.0 International license.

### Genes impacting S. gordonii fitness.

**FIG 2:**
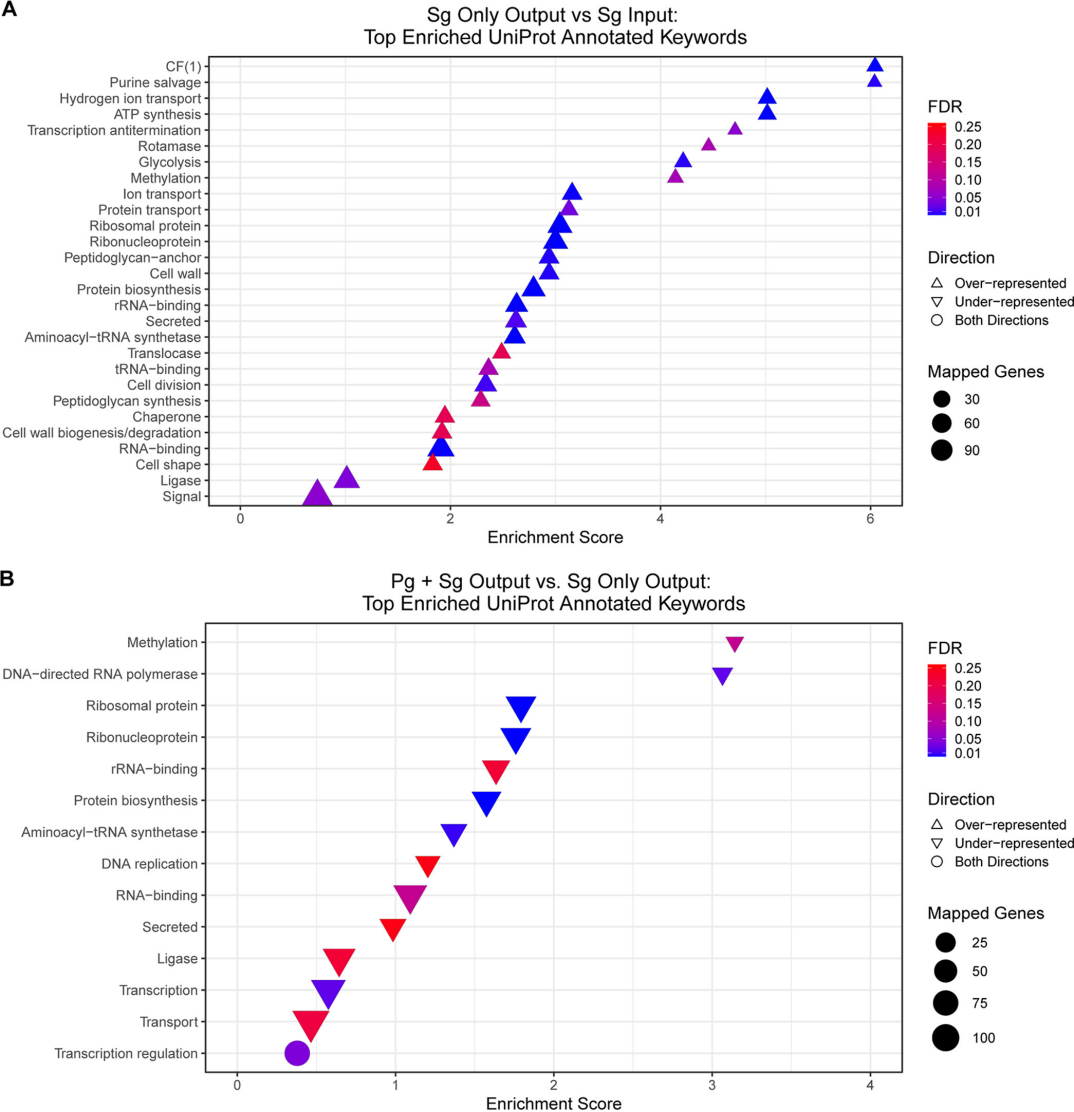
Functional analysis of S. gordonii (Sg) genes showing fitness effects in monoinfection (A) or coinfection with P. gingivalis (Pg) (B). UniProt keywords that are functionally enriched by *S. gordonii* genes showing fitness effects are shown. Functional enrichment analysis was performed via STRING v.11.5 using the “Proteins with Values/Ranks - Functional Enrichment Analysis” function with an FDR stringency of 25% and a minimum-interaction confidence score of 0.4 for network generation.

Complete lists of the genes exhibiting differential fitness are given in Table S1. A pathway enrichment analysis using the STRING database showing the top enriched UniProt keywords under the Sg-alone and Sg+Pg conditions is shown in Fig. 2. Comparison of the Sg-alone output with the input library revealed a broad range of pathways that had an impact on fitness. Interestingly, by this analysis, all the pathways were overrepresented in the output pool and thus were nonessential for S. gordonii survival *in vivo*. Hence, nutrients or necessary cofactors provided *in vivo* may provide considerably more support to S. gordonii growth and survival compared to laboratory media, resulting in a number of pathways dispensable *in vivo*. Under the Sg+Pg condition, compared to Sg-alone, a more limited range of pathways was associated with fitness effects, predominantly representing pathways that were essential *in vivo*. A number of these were related to DNA replication, transcription, translation, and transport, indicating that robust growth in the dual-species abscess is required for S. gordonii to remain competitive. Indeed, the extensive nature of changes in CoDE genes in S. gordonii in the context of a coinfection with P. gingivalis, compared to a monospecies infection, is indicative of extensive metabolic integration of the two species. Oral bacteria participate in extensive trophic networks (34, 35), and P. gingivalis can increase the levels of S. gordonii proteins comprising glycolysis and pentose phosphate pathways (29). While S. gordonii CoDE genes may have direct relevance to the pathogenicity of P. gingivalis-S. gordonii communities, as they span a range of broadly based metabolic functions without a clear overall theme, they were not addressed further in this study.

A fitness score was calculated for each gene as the log_2_ difference in abundance in the coinfection group relative to the monoinfection group. We averaged the fitness score for each gene across all 5 biological replicates and defined a strong fitness effect as a score of >1 (either positive or negative) with an adjusted *P* value of <0.05. Interrogation of the genes associated with increased fitness (i.e., mutants were overrepresented in the output pool) of the Sg+Pg coinfection condition identified a cluster of genes in the receptor polysaccharide (RPS) operon. The RPS operon was originally identified as mediating the production of extracellular polysaccharides that participate in coadherence with *Actinomyces* species (36). However, S. gordonii strain Challis does not bind to *Actinomyces*, and RPS may also be involved in interactions with host cells and tissues (37, 38). To investigate this system further, we first examined the S. gordonii strain Challis CH1 operon by reverse transcription-PCR (RT-PCR) (Fig. S2) and found that it extends from SGO_2039 to SGO_2012. As shown in Table 1, 13 of these genes showed a strong fitness effect, whereby mutation in the gene increased survival during coinfection with P. gingivalis. Moreover, 8 of the genes were conditionally essential in the Sg-alone output (Table S1B), indicating that P. gingivalis in the community context rescued the fitness defect resulting from loss of gene function. Four genes showed a slight decrease in fitness scores; however, none of these was less than −1. Three genes are not represented as they had a total count across all samples of less than 10. In addition, analysis of mRNA levels by quantitative RT-PCR (qRT-PCR) showed that expression of SGO_2020 and SGO_2024 was reduced in CH1 upon adaptation to a community infection with P. gingivalis (Fig. S3A), further evidence that expression of these genes is disadvantageous to S. gordonii survival in the presence of P. gingivalis.

**TABLE 1:** Coinfection fitness scores of genes in the RPS operon of S. gordonii

Gene no.	Annotation	Fitness score^*a*^	Adjusted *P* value
SGO_2012	Transmembrane protein	−0.23	8.44E−01
SGO_2013	Putative *N*-acetylmuramidase	1.02	1.42E−05
SGO_2014	Transposase, ISL3 family, degenerate	4.1	1.93E−03
SGO_2015	Putative polysaccharide transport protein	3.76	3.9E−12
SGO_2016	Putative nucleotide sugar dehydratase	0.33	5.5E−01
SGO_2017	*ispD*, ribitol-5-phosphate cytidylyltransferase	3.50	9.4E−27
SGO_2018	Putative extracellular polysaccharide polymerase	2.36	3.4E−29
SGO_2019	LicD3 protein	2.91	8.1E−25
SGO_2020	Glycosyltransferase	3.30	1.4E−21
SGO_2021	Extracellular polysaccharide glycosyltransferase	1.17	2.6E−10
SGO_2022	UDP-glucose 4-epimerase	1.12	4.0E−11
SGO_2023	Galactosyltransferase	0.91	7.7E−06
SGO_2024	Extracellular polysaccharide biosynthesis	2.32	3.7E−18
SGO_2025	*wze*, tyrosine-protein kinase	1.05	9.2E−12
SGO_2026	*wzd*, capsular polysaccharide biosynthesis protein	0.15	5.5E−01
SGO_2027	*wzh*, tyrosine-protein phosphatase,	3.24	6.3E−29
SGO_2028	*wzg*, transcriptional regulator, LytR family	−0.05	7.7E−01
SGO_2029	Anaerobic ribonucleoside-triphosphate reductase-activating protein	−0.61	3.0E−01
SGO_2030	Acetyltransferase, GNAT family	0.78	8.3E−05
SGO_2031	Acetyltransferase, GNAT family	0.86	2.7E−05
SGO_2033	*nrdD*, ribonucleoside-triphosphate reductase	−0.36	1.2E−02
SGO_2034	Putative membrane protein	0.61	3.2E−02
SGO_2037	Cardiolipin synthase	1.16	3.8E−10
SGO_2038	Putative lipoprotein	0.14	1.5E−01
SGO_2039	Dihydrofolate synthase	0.86	1.6E−06

a Log_2_ fold change of gene expression values between the Sg+Pg group and Sg-only group. Positive numbers indicate gene mutants whose fitness enhanced by the presence of *P. gingivalis*. Negative numbers indicate gene mutants whose fitness was decreased by the presence of *P. gingivalis*.

10.1128/mbio.00658-23.2 FIG S2 RPS operon. PCR was used to investigate the operon arrangement of the RPS-encoding genes in S. gordonii. Cells were disrupted using TRIzol and a FastPrep-24 5G (MP Biomedicals). RNA was isolated using an RNeasy kit (Qiagen), and genomic DNA (gDNA) contamination was removed with a TURBO DNA-free kit (Invitrogen). A total of 2 μg RNA was converted to cDNA using a high-capacity cDNA synthesis kit (Applied Biosystems). Primers (A to O) are listed in Table S2, and positions along with predicted product size are indicated in the gene cluster diagram (A). RT-PCR, PCR with gDNA, and a control with no reverse transcriptase (RT) are shown by agarose gel electrophoresis in panel B. The arrows in panel A indicate the leading gene (SGO_2039) and terminal gene (SGO_2012) of the operon. Download FIG S2, PDF file, 0.3 MB. Copyright © 2023 Pandey et al. 2023 Pandey et al. https://creativecommons.org/licenses/by/4.0/ This content is distributed under the terms of the Creative Commons Attribution 4.0 International license.

10.1128/mbio.00658-23.3 FIG S3 (A) qRT-PCR of SGO_2010 (outside the RPS operon), SGO_2020, and SGO_2024 mRNA expression in S. gordonii isolated from either S. gordonii alone or S. gordonii plus P. gingivalis abscess material. cDNA was prepared as described in the Fig. S2 legend. mRNA levels were normalized to 16S RNA and expressed relative to S. gordonii alone. Data are means ± SD from 4 biological replicates. *, *P* < 0.05 by Mann-Whitney U test. (B) Confirmation of nonpolar mutations in the Δ2020 and Δ2024 mutants. mRNA extraction and cDNA synthesis were performed as described in the Fig. S2 legend. qRT-PCR was performed for the genes downstream of the deleted genes within the RPS operon (B) and a gene (SGO_2010) downstream of the RPS operon (C). Values are expression levels relative to the parental S. gordonii CH1 strain (WT). Primers are listed in Table S2. (D) Gram staining of S. gordonii CH1 (WT) and the Δ2020 and Δ2024 mutants following recovery from mouse abscesses, *in vitro* culture, or *in vitro* culture following vortexing for 10 s. Download FIG S3, PDF file, 4.3 MB. Copyright © 2023 Pandey et al. 2023 Pandey et al. https://creativecommons.org/licenses/by/4.0/ This content is distributed under the terms of the Creative Commons Attribution 4.0 International license.

Interestingly, two putative genes in the operon are on the opposite strand, namely, SGO_2014 and SGO_2035. While SGO_2035 potentially encodes a 32-amino-acid (aa) hypothetical protein, and was not represented in the input pool (Table S1A), SGO_2014 showed a strong fitness effect. The protein encoded by SGO_2014 is annotated as a transposase, which may have relevance to the acquisition of the operon. Additionally, these data indicate that the RPS operon constitutes a member of an emerging class of noncontiguous bacterial operons that contain genes transcribed in the opposite direction to the rest of the operon (39).

### Strains with mutations in the RPS operon.

**FIG 3:**
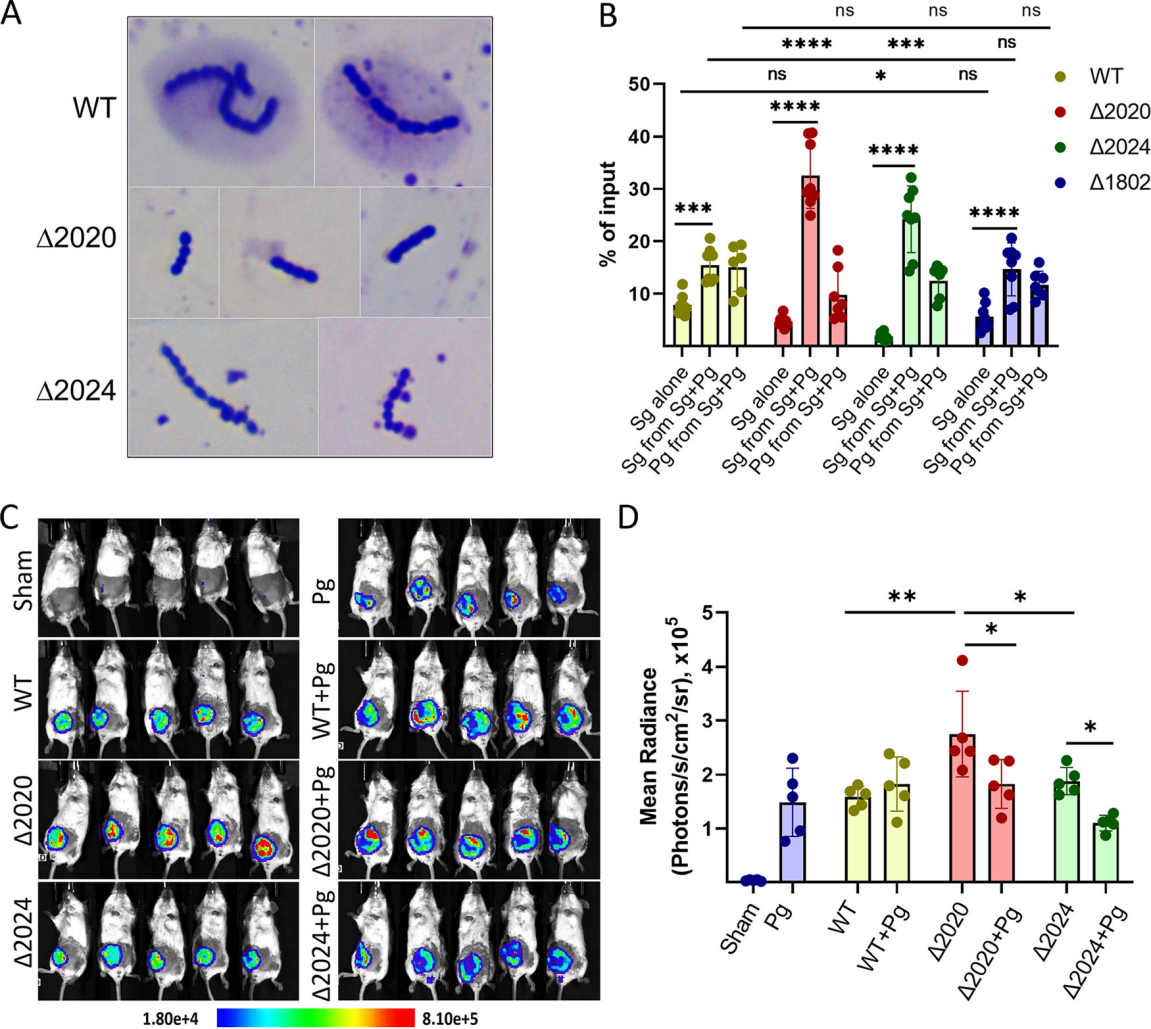
P. gingivalis rescues RPS mutants *in vivo* and diminishes neutrophil mobilization. (A) Extracellular polysaccharide production by S. gordonii CH1 (WT), Δ2020, and Δ2024 strains visualized by Anthony’s stain; (B) recovery of P. gingivalis (Pg) or of S. gordonii (Sg) CH1 (WT), Δ2020, Δ2024, or Δ1802 cells from mouse abscesses with or without *P. gingivalis*. Data are from 8 mice. S. gordonii alone was inoculated at 3 × 10^9^ CFU or together with P. gingivalis at 1.5 × 10^9^ CFU each. Percentage of recovery was calculated based on the total bacterial input number. (C) IVIS imaging of mice inoculated subcutaneously (s.c.) with strains as indicated and after 4 h injected intraperitoneally (i.p.) with IVISbrite MPO chemiluminescent probe to detect myeloperoxidase activity of neutrophils. The scale bar shows radiance in photons per second per square centimeter per steradian. (D) Plot of radiance in panel C as mean ± standard deviation (SD) from each group. *, *P* < 0.05; **, *P* < 0.01; ***, *P* < 0.005; and ****, *P* < 0.001; by two-way analysis of variance (ANOVA) with Sidak’s multiple-comparison test. ns, not significant.

In order to investigate the role of the RPS operon in S. gordonii-P. gingivalis coinfections, we created mutants with deletion of SGO_2020 (Δ2020), coding for a putative glycosyltransferase, and SGO_2024 (Δ2024), coding for a putative epimerase. RT-PCR confirmed loss of the relevant mRNA in these mutants without a reduction in the expression of downstream genes, either within or outside the operon, indicating nonpolar mutations (Fig. S3B and C). Anthony’s stain demonstrated loss of extracellular polysaccharide material in both the mutants (Fig. 3A). Additionally, both mutants formed longer chains when cultured *in vitro*, but not when recovered from abscesses (Fig. S3D). Vortexing reduced the chain length to parental levels (Fig. S3D), and for subsequent *in vitro* assays, suspensions of bacterial cells were vortexed prior to use. Moreover, the mutants supported P. gingivalis coadherence to a greater extent than the parental strain (Fig. S4). This result aligns with the reported coadherence specificity of RPS for actinomyces and also indicates that the RPS may impede access of P. gingivalis fimbrial adhesins to their protein receptors on the streptococcal surface (40). We attempted to complement the SGO_2020 mutant using the wild-type (WT) allele in *trans*; however, although mRNA was expressed, restoration of polysaccharide production or chain length did not occur (Fig. S5), indicating posttranscriptional requirements were not met. Similarly, Kovacs et al. (41) were unable to genetically complement mutants in cell wall polysaccharide in Streptococcus mutans.

10.1128/mbio.00658-23.4 FIG S4 Community formation of P. gingivalis with S. gordonii. P. gingivalis (CFSE labeled [green]) was reacted with a substratum of the S. gordonii WT strain or Δ2020 and Δ2024 mutants (hexidium iodide labeled [red]) for 18 h and imaged by confocal microscopy. (A) Ratio of P. gingivalis to S. gordonii biovolume from 6 random fields. Data are means ± SD. *, *P* < 0.05; and **, *P* < 0.01; using ANOVA with Tukey's multiple-comparisons test. (B and C) *x-z* and *x-y* projections of reconstructed images using Volocity software. The size bar represents 10 μm. Download FIG S4, PDF file, 0.6 MB. Copyright © 2023 Pandey et al. 2023 Pandey et al. https://creativecommons.org/licenses/by/4.0/ This content is distributed under the terms of the Creative Commons Attribution 4.0 International license.

10.1128/mbio.00658-23.5 FIG S5 Complementation of the Δ2020 mutant with SGO_2020 in *trans*. The strains were S. gordonii CH1 (WT), the Δ2020 mutant, the Δ2020 mutant complemented in *trans* with plasmid pVA749 containing SGO_2020, along with the S. mutans *ldh* promoter upstream of the native putative ribosomal binding site and ATG start; or the Δ2020 mutant containing empty pVA749 vector. (A) Gram-staining; (B) growth in BHI medium; (C) qRT-PCR for SGO_2020 mRNA (primers in Table S2); (D) RPS production visualized by Anthony’s stain. Although the complemented mutant produced SGO_2020 mRNA, phenotypic complementation was not achieved. Download FIG S5, PDF file, 0.3 MB. Copyright © 2023 Pandey et al. 2023 Pandey et al. https://creativecommons.org/licenses/by/4.0/ This content is distributed under the terms of the Creative Commons Attribution 4.0 International license.

The total glycosyl composition of soluble cell wall polysaccharides of mutant and WT strains was analyzed. We found a significant decrease in cell wall GalNAc contents of both the Δ2020 and Δ2024 mutants (Table 2), consistent with previous reports showing that the majority of RPS types characterized contain GalNAc as part of their backbone structure (42). Additionally, the RPSs in S. gordonii 38 and in Streptococcus oralis J22 both contain rhamnose branches that form the major antigenic determinants (42). There was a significant decrease in extracellular rhamnose content of both the Δ2020 and Δ2024 mutants, suggesting that rhamnose branching may also play a role in determining the antigenic specificity of CH1. Ribose concentrations were also decreased and glucose was proportionally higher in the mutants. Taken together, these results confirm major changes in cell polysaccharide composition in the Δ2020 and Δ2024 mutants.

**TABLE 2:** Glycosyl composition of polysaccharide extracts recovered from S. gordonii strains

Strain	Glycosyl residue (mol %)					
	Rib	Rha	Glc	GlcNAc	GalNAc	MurNAc
WT CH1	8.5	63.0	1.7	0.1	26.1	0.6
Δ2020 mutant	0	33.7	53.1	0.0	12.9	0.3
Δ2024 mutant	0	18.3	74.0	0.0	7.5	0.2

We also examined the protein profiles and antigenic characteristics of lysates of S. gordonii WT and mutant strains by SDS-PAGE and by blotting with antibodies raised to formalin-fixed whole S. gordonii cells. Figure S6 shows similar banding patterns among the strains, in terms of both total proteins and immunogenic surface proteins. Additionally, there was no difference in susceptibility of mutant strains to the peptidoglycan-targeting antibiotic penicillin (Fig. S7A), further validating the production of RPS as the major difference between WT and mutants, with no impairment of peptidoglycan synthesis or expression of surface proteins.

10.1128/mbio.00658-23.6 FIG S6 Protein expression and antigenic profile in S. gordonii strains. Bacteria were grown in BHI broth to OD_600_ of ~0.8. Equal amounts of cells were harvested, washed, and resuspended in a mixture of 50 mM Tris-Cl (pH 7.6), 150 mM NaCl, and 5% glycerol. Cells were mechanically disrupted using a FastPrep-24 5G. The lysate was centrifuged at 10,000 rpm for 5 min, and the pellet was dissolved in SDS-PAGE sample buffer, separated by SDS-PAGE on a 12% gel, and stained with Coomassie brilliant blue (A). For Western blotting (B), proteins were electrotransferred (40 V, 2 h) from unstained gels onto polyvinylidene difluoride (PVDF) membranes. The membrane was blocked with 4% skimmed milk for 1 h, and washed 3 times in TBST (TBS with 0.05% Tween 20) for 10 min. Primary rabbit antibody raised to S. gordonii strain G9B at 1:5,000 was reacted overnight at 4°C, followed by being washed again with TBST. The secondary antibody was anti-rabbit IgG conjugated with horseradish peroxidase (HRP) (1:2,000) and was reacted for 1 h at 4°C. The membrane was washed and developed using chemiluminescent substrate (SuperSignal West PicoPlus; Thermo Scientific). Images are representative of 3 biological replicates. Download FIG S6, PDF file, 0.3 MB. Copyright © 2023 Pandey et al. 2023 Pandey et al. https://creativecommons.org/licenses/by/4.0/ This content is distributed under the terms of the Creative Commons Attribution 4.0 International license.

10.1128/mbio.00658-23.7 FIG S7 (A) Penicillin sensitivity of S. gordonii strains. The minimum inhibitory concentration (MIC) was determined for penicillin (Sigma) using the broth microdilution method. Values are means ± SD. (B) Representative Amnis Imagestream images of WGA-stained human neutrophils (red) reacted for 30 min with CFSE-stained S. gordonii strains (green) with or without P. gingivalis as indicated. “US” indicates unstimulated cells. (C) Plot of CFSE fluorescence associated with WGA-stained neutrophils (shown in panel B) from 60 cells; (D) induction of reactive oxygen species (ROS). Human neutrophils were challenged with S. gordonii strains with or without P. gingivalis at the MOIs indicated at 37°C or were left unstimulated (Unstim). Total (D) and intracellular (E) ROS measured by lucigenin-elicited chemiluminescence. Intracellular ROS was detected in the presence of superoxide dismutase (SOD) to remove extracellular ROS. Summed integrated responses measured as relative light units (RLU) per second recorded over 1 h are shown. (F) Viability of P. gingivalis from human neutrophils after coinfection with S. gordonii. Neutrophils were challenged with P. gingivalis and S. gordonii strains for 20 min in 5% CO_2_ each at an MOI of 10. Colony-forming units (CFU) were calculated from postlysis solutions incubated on TSB agar plates anaerobically. (G) Degranulation of elastase from human neutrophils. P. gingivalis cells were either pretreated with 100 μM of TLCK for 2 h at 37°C anaerobically or left untreated. (H) Elastase release after infection with P. gingivalis alone at the MOIs indicated for 4 h. “Stim” is stimulated with latrunculin (1 μM) for 30 min and fMLF (1 μM) for 10 min. Data are means ± SD. *, *P* < 0.05; **, *P* < 0.01; ***, *P* < 0.05; and ****, *P* < 0.001; using 2-way ANOVA with Tukey's multiple-comparisons test. ns, not significant. Download FIG S7, PDF file, 3.3 MB. Copyright © 2023 Pandey et al. 2023 Pandey et al. https://creativecommons.org/licenses/by/4.0/ This content is distributed under the terms of the Creative Commons Attribution 4.0 International license.

### *In vivo* survival of Δ2020 and Δ2024 mutants and neutrophil mobilization.

To corroborate the results of the Tn library screen, we tested the survival of isogenic SGO_2020 and SGO_2024 deletion mutants in the *in vivo* abscess model (Fig. 3B). The Δ2024 mutant displayed a slight fitness defect in the context of a monoinfection. In contrast, the presence of P. gingivalis increased the survival of both Δ2020 and Δ2024 mutants by over 7-fold. Additionally, P. gingivalis also increased the survival of the WT S. gordonii strain 2-fold, showing synergism between the two species, which is enhanced when S. gordonii lacks RPS. As a control, a mutant with a deletion in SGO_1802 (Δ1802), encoding a putative metal ion uptake system component, was tested. Loss of SGO_1802 did not show a fitness effect in the abscess model. P. gingivalis increased survival of the Δ1802 mutant to the same extent as the WT strain and less than the Δ2020 and Δ2024 mutants. No difference in the survival rates of P. gingivalis with the different streptococcal strains was observed. Collectively, these results indicate P. gingivalis cooperates with S. gordonii to increase *in vivo* survival of the streptococci, and this effect is more pronounced when S. gordonii does not produce RPS.

As neutrophils constitute a predominant host immune cell population in acute abscesses, we examined neutrophil mobilization in response to infection. IVIS imaging using a chemiluminescent myeloperoxidase (MPO)-reactive dye showed that S. gordonii WT and P. gingivalis alone, as well as the Δ2020 and Δ2024 mutants, induced neutrophil recruitment, with elevated responses to the Δ2020 mutant compared to the WT and Δ2024 mutant (Fig. 3C and D). In the coinfection context, P. gingivalis reduced neutrophil mobilization in response to both the Δ2020 and Δ2024 mutants, but not to the WT. Taken together these data suggest that P. gingivalis can enhance the survival of S. gordonii *in vivo*, in part by modulating neutrophil mobilization, which then obviates the need for production of energetically costly extracellular polysaccharides.

### Opsonization and phagocytosis of Δ2020 and Δ2024 mutants.

**FIG 4:**
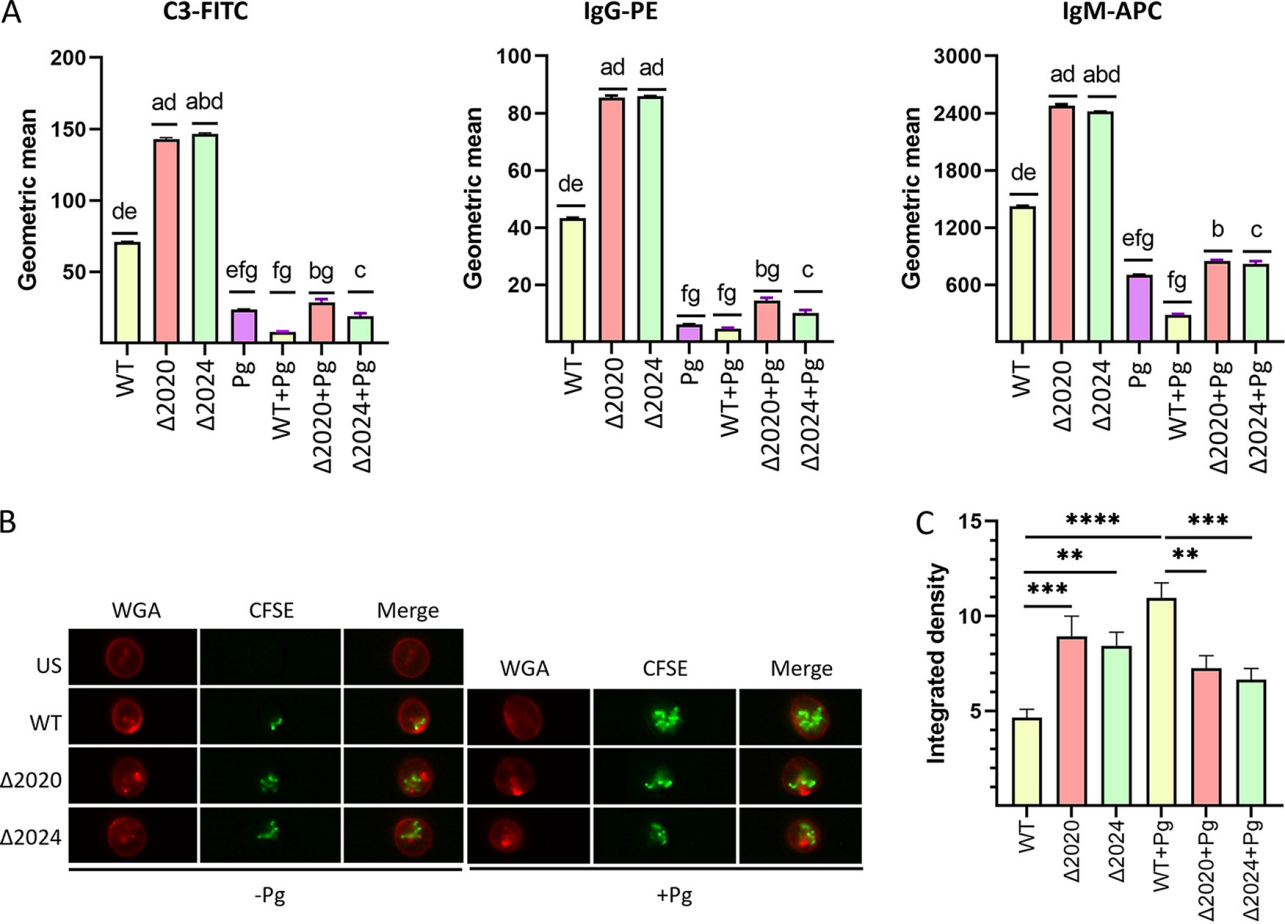
Opsonization and phagocytosis of S. gordonii strains. (A) Opsonization of S. gordonii strains (1 × 10^9^ CFU) with or without P. gingivalis (Pg) (1 × 10^9^ CFU) as indicated was performed in 10% human serum. Bacteria were probed with fluorophore-conjugated antibodies (C3-FITC, IgG-PE, and IgM-APC) and analyzed by flow cytometry. Data are geometric means ± SD of fluorescence, and the statistical differences between the groups were calculated using two-way ANOVA with Tukey's multiple-comparison test. The letters a to g indicate statistical difference (*P* < 0.05) comparisons as follows: a to WT, b to the Δ2020 mutant, c to the Δ2024 mutant, d to *P. gingivalis*, e to WT plus *P. gingivalis*, f to Δ2020 mutant plus *P. gingivalis*, and g to Δ2024 mutant plus *P. gingivalis*. (B) Representative Amnis Imagestream images of WGA-stained human neutrophils (red) reacted for 30 min with serum-opsonized CFSE-stained S. gordonii strains (green) with or without P. gingivalis as indicated. “US” indicates unstimulated cells. (C) Plot of CFSE fluorescence associated with WGA-stained neutrophils from 60 cells. Values are means ± standard error (SE). **, *P* < 0.01; ***, *P* < 0.005; and ***, *P* < 0.001; using two-way ANOVA with Tukey's multiple-comparison test.

The ability of the Δ2020 and Δ2024 mutants to be opsonized by human serum was tested using flow cytometry. Compared to the WT strain, binding of C3, IgG, and IgM opsonins in the absence of RPS was elevated (Fig. 4A). In the presence of P. gingivalis, however, opsonization of WT and the Δ2020 and Δ2024 mutants was significantly reduced. Imaging flow cytometry was then used to determine the susceptibility of S. gordonii strains to neutrophil phagocytosis. Consistent with the opsonization results, internalization of carboxyfluorescein succinimidyl ester (CFSE)-labeled serum-opsonized Δ2020 and Δ2024 cells was higher than WT levels (Fig. 4B and C). However, the presence of P. gingivalis had no statistically significant effect on Δ2020 and Δ2024 phagocytosis and, surprisingly, increased phagocytosis of WT cells, indicating P. gingivalis can have an opsonin-independent effect on WT phagocytosis. Comparison of the phagocytosis of unopsonized cells also showed that P. gingivalis can increase the internalization of WT S. gordonii, without affecting uptake of the Δ2020 and Δ2024 mutants (Fig. S7B and C). Similar to opsonized cells, phagocytosis of unopsonized Δ2020 and Δ2024 cells was higher than that of the WT. Thus, P. gingivalis can enhance the phagocytosis of S. gordonii when the RPS is present, while loss of RPS allows neutrophils to phagocytose S. gordonii more efficiently, independent of opsonization. The finding that P. gingivalis does not inhibit phagocytosis of the Δ2020 and Δ2024 mutants suggested that interference with intracellular neutrophil killing mechanisms may occur.

### Neutrophil survival of Δ2020 and Δ2024 mutants.

**FIG 5:**
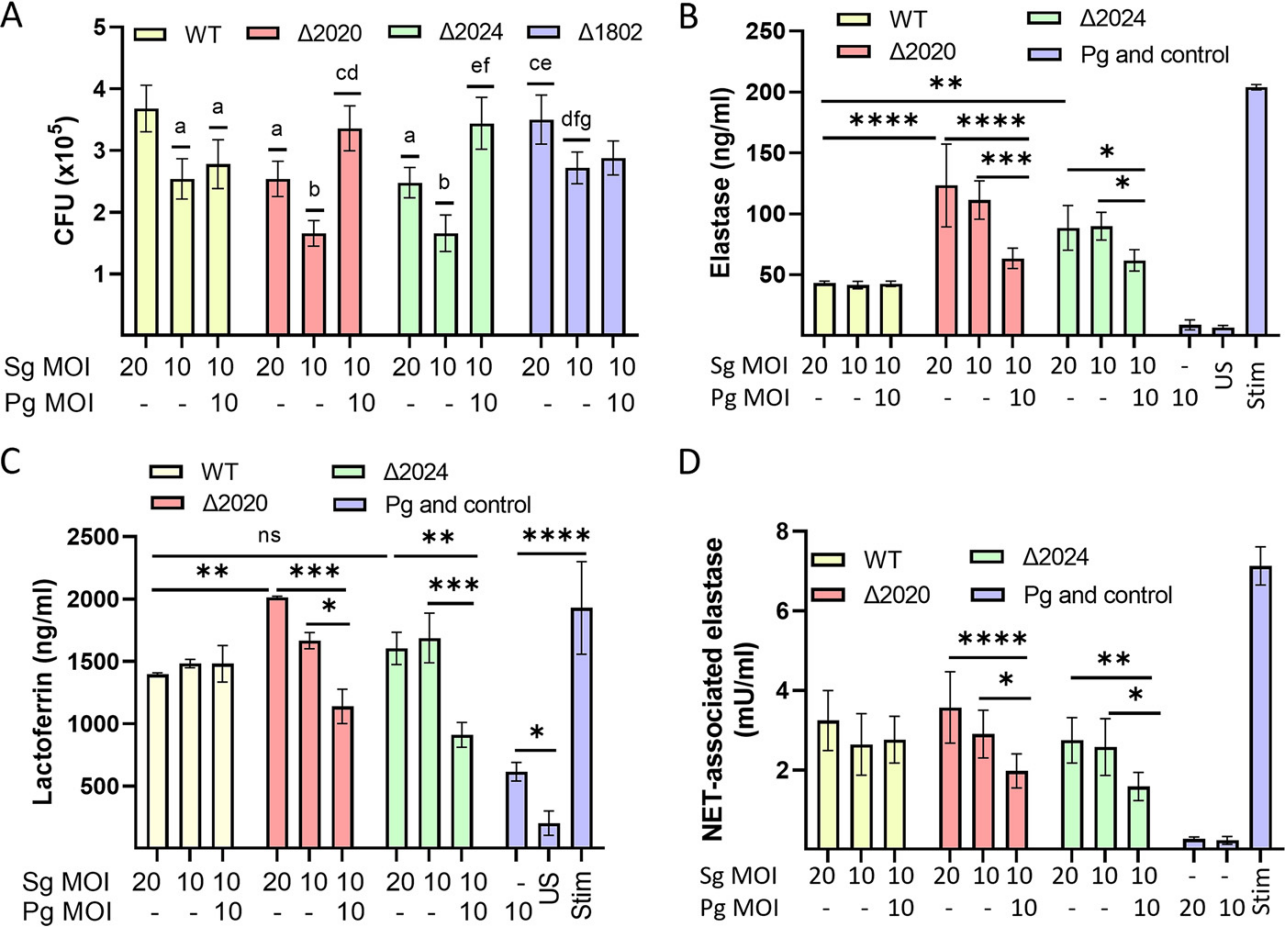
P. gingivalis rescues Δ2020 and Δ2024 mutants from neutrophil killing *in vitro* by diminishing elastase and lactoferrin responses. (A) Neutrophil killing of S. gordonii WT and the Δ2020, Δ2024, or Δ1802 mutants with or without P. gingivalis (Pg) at the multiplicity of infection (MOI) indicted. CFU represent bacteria cultured from a postlysis solution of human neutrophils. Values are means ± SD. The letters a to g’ indicate statistical difference (*P* < 0.05) comparisons as follows: a to WT with MOI of 20, b to WT with MOI of 10, c to Δ2020 mutant with MOI of 20, d to Δ2020 mutant with MOI of 10, e to Δ2024 mutant with MOI of 20, f to Δ2024 mutant with MOI of 10, and g to Δ1802 mutant with MOI of 20 using two-way ANOVA with Tukey's multiple-comparison test. (B) Degranulation of elastase, (C) degranulation of lactoferrin, and (D) NET formation in neutrophils challenged with S. gordonii strains with or without P. gingivalis at the MOIs indicated. “US” is unstimulated, and “Stim” is stimulated with latrunculin (1 μM) for 30 min and fMLF (1 μM) for 10 min (B and C) or PMA (20 nM) for 4 h (D). Values are means ± SD. *, *P* < 0.05; **, *P* < 0.01; ***, *P* < 0.005; and ****, *P* < 0.001; using two-way ANOVA with Tukey's multiple-comparison test. ns, not significant.

To further examine cooperativity between P. gingivalis and S. gordonii in evading neutrophil-mediated immune protection, we tested resistance to neutrophil killing. Decreased viability of the Δ2020 and Δ2024 mutants compared to S. gordonii WT was observed (Fig. 5A). While P. gingivalis did not affect survival of S. gordonii WT within neutrophils, survival of the Δ2020 and Δ2024 mutants was enhanced by coinfection with P. gingivalis at a multiplicity of infection (MOI) of 10 for both species (Fig. 5A). This effect was not caused by higher numbers of bacteria under the coinfection condition compared to S. gordonii alone as the coinfection condition showed higher levels of survival compared to Δ2020 and Δ2024 mutants alone at an MOI of 20. In contrast, survival of the Δ1802 mutant was not increased by coinfection with P. gingivalis (Fig. 5A), and there was no difference in the survival of P. gingivalis (Fig. S7F).

### Resistance to neutrophil killing mechanisms.

One of the early and efficient antimicrobial responses of neutrophils is the robust generation of reactive oxygen species (ROS) through the assembly and activation of the NADPH oxidase multisubunit enzyme. As shown in Fig. S7D and E, the Δ2020 and Δ2024 mutants induced more intracellular and total ROS production than the WT, consistent with higher levels of internalization of the mutant strains. However, oral streptococci are generally resistant to killing by ROS (30), and ROS production was not inhibited by P. gingivalis, indicating that synergy between P. gingivalis and the Δ2020 and Δ2024 mutants does not involve ROS production.

Release of elastase from azurophilic granules and release of lactoferrin from specific granules are additional antibacterial mechanisms of neutrophils (43, 44). As shown in Fig. 5B, the Δ2020 and Δ2024 mutants incited higher elastase release than the WT strain. Moreover, coinfection with P. gingivalis resulted in a significant reduction in elastase release. The effect of P. gingivalis was not dependent on gingipain activity as the gingipain inhibitor TLCK (*N*α-*p*-tosyl-l-lysine chloromethyl ketone) did not prevent inhibition of elastase release (Fig. S7G and H). Similarly, while P. gingivalis alone elicited a small but significant increase in lactoferrin degranulation, P. gingivalis suppressed lactoferrin degranulation in response to the Δ2020 and Δ2024 mutants (Fig. 5C). Additionally, neutrophils can kill bacteria through a process defined as neutrophil extracellular trap (NET) formation (45). NETs are DNA structures containing granular antimicrobial proteins, such as myeloperoxidase (MPO), neutrophil elastase (NE), and citrullinated histones. While the WT and mutant strains stimulated similar levels of NETs (Fig. 5D), in the presence of P. gingivalis, NET formation with the Δ2020 and Δ2024 mutants was diminished. Collectively, these results show that P. gingivalis can inhibit elastase and lactoferrin release as well as NET formation induced by RPS-deficient S. gordonii mutants and this may represent a means by which P. gingivalis can protect Δ2020 and Δ2024 mutants from neutrophil killing.

## DISCUSSION

P. gingivalis and S. gordonii engage in dynamic and multidimensional interactions, whereby either can alter the metabolic status of the other (1, 13, 46). These interactions can also modulate the nososymbiocity of the community, and indeed, in a murine model, P. gingivalis can instigate the transition of communities from homeostatic to dysbiotic (14). Pathogenesis at mucosal barriers, or at tissues following systemic spread, requires that the community evade host immune responses and survive *in vivo*. Coaggregation between P. gingivalis and Fusobacterium nucleatum has been shown to impede neutrophil phagocytosis (47), while P. gingivalis can augment macrophage phagocytosis of Tannerella forsythia in a gingipain-dependent manner (48). It has also been shown that P. gingivalis coactivates TLR2 (Toll-like receptor 2) and C5aR in neutrophils, and the resulting cross talk leads to proteasomal degradation of MyD88, thereby inhibiting antimicrobial responses (49). Additionally, C5aR-TLR2 cross talk activates phosphatidylinositol 3-kinase (PI3K), which prevents phagocytosis and thus protects community partners such as F. nucleatum (49). In macrophages, P. gingivalis can repress inflammasome responses to F. nucleatum by reducing the level of endocytosis (50). However, little is known regarding interspecies cooperation between P. gingivalis and antecedent colonizing streptococci and the impact on *in vivo* survival. In this study, we used a Tn-Seq library of S. gordonii to identify components that contribute to *in vivo* fitness in the context of a community with P. gingivalis.

In an abscess model, coinfection with P. gingivalis increased the survival of S. gordonii, demonstrating that the two organisms exhibit polymicrobial synergy that impacts *in vivo* fitness. We found CoDE genes required for fitness with P. gingivalis included several transcription factors as well as genes involved in DNA replication, translation, and transport. Remarkably, the conditionally essential requirement of genes in the monoinfection context was almost completely alleviated by P. gingivalis. This result is suggestive of extensive metabolic interactions between P. gingivalis and S. gordonii, dramatically altering gene essentiality during polymicrobial infection. Similar trends have been reported with Staphylococcus aureus in a coinfection with Pseudomonas aeruginosa (32) and Aggregatibacter actinomycetemcomitans in a coinfection with S. gordonii (10). Conditional gene essentiality of bacteria in communities, therefore, would appear to be highly dependent on context. For example, a comparison of the P. gingivalis genes essential for coinfection with S. gordonii or with Fusobacterium nucleatum revealed only two in common (31). Community partner-dependent changes in fitness determinants reinforce the basis of current concepts of periodontal disease etiology, whereby certain species, even in low abundance, can have a major influence on community pathogenicity (51).

In addition to the community-dependent essential genes, Tn-Seq data sets reveal gene insertions that are overrepresented in the community output pool, a category not always fully considered in Tn-Seq studies. In contrast to the diffuse nature of functions contributed by S. gordonii genes conditionally essential in a coinfection with P. gingivalis, many of the mutations that were detrimental to S. gordonii fitness but rescued with P. gingivalis were in genes involved in the production of receptor polysaccharide (RPS). Indeed, 13 genes of a 28-gene operon, extending beyond the region originally proposed (52), were significantly detrimental to the fitness of S. gordonii in coinfection with P. gingivalis. Many strains of S. gordonii and related oral streptococci produce extracellular RPSs, the structure and function of which depend on the combinations of transferases and polymerases (36, 53). In some, but not all, strains, streptococcal RPSs are involved in coadhesion to other bacteria, while the role of RPSs in other functions, including regulation of chain length or protection against host immune responses has not been investigated. Mutation of SGO_2020 or SGO_2024 resulted in a change in the composition of soluble polysaccharides and in loss of extracellular polysaccharide as observed by Anthony’s stain, confirming their involvement in synthesis of RPS. SGO_2020 is annotated as coding for a glycosyltransferase, an enzyme that catalyzes the biosynthesis of glycosidic bonds in oligosaccharides, polysaccharides, and glycoconjugates by transferring a saccharide moiety from a nucleotide sugar donor to a nucleophilic glycosyl acceptor. SGO_2024 codes for a putative epimerase that will invert the hydroxyl substituents on carbohydrates. Loss of RPS also resulted in increased chain length of cells cultured *in vitro*, similar to observations in S. mutans lacking the rhamnose-glucose polysaccharide (41). Interestingly, increased chain length of the Δ2020 and Δ2024 mutants was not observed in cells recovered from abscesses, indicating that conditions *in vivo* can override the role of RPS in cell division. It has also been shown that fatty acids can impact streptococcal chain length (54), and thus concentrations of specific fatty acids in the abscess environment may be the determining factor in this phenomenon.

Given that capsule/extracellular polysaccharide in other organisms can contribute to resistance to oxidative and acidic stress, opsonization, phagocytosis, and killing, we postulated that in the absence of RPS, bacteria are more easily targeted by the immune system. We further proposed that P. gingivalis is able to compensate for loss of these functions, and hence in the presence of P. gingivalis, production of energetically costly RPS is a disadvantage to S. gordonii survival. In support of this concept, the presence of P. gingivalis diminished the ability of human neutrophils to kill the mutants lacking RPS. The gingipain proteases of P. gingivalis can degrade antibody and complement opsonins (55, 56), and consistent with this, we found that P. gingivalis reduced neutrophil mobilization in the mouse abscess model and decreased the level of streptococcal cell binding by C3, IgG, and IgM opsonins. Interestingly, however, while degradation of opsonins has been shown to decrease the phagocytosis of P. gingivalis (57), and opsonization enhanced neutrophil phagocytosis of S. gordonii, as also reported by others (58), in the current study, we did not observe a reduction in phagocytosis of opsonized RPS mutants in the presence of P. gingivalis. Residual levels of opsonins may therefore be sufficient to maintain phagocytosis levels. Additionally, there was a slight increase in the phagocytosis of the WT strain with P. gingivalis, possibly due to the formation of aggregates which can be more easily phagocytosed (59).

A major synergistic effect of P. gingivalis was on the production of neutrophil antimicrobial factors induced by the RPS mutants, in particular the inhibition of elastase and lactoferrin degranulation. Indeed, P. gingivalis at an MOI of 10 did not stimulate the release of elastase from granules, which may require higher bacterial numbers and a longer time of exposure (60, 61). Neutrophil elastase can directly kill Gram-negative bacteria, such as Klebsiella pneumoniae and Escherichia coli, as well as some Gram-positive bacteria, such as S. pneumoniae, particularly in combination with cathepsin G and proteinase 3 (62, 63). Elastase degranulation can also lead to the extrusion of neutrophil NETs, web-like chromatin structures that can trap, neutralize, and kill microorganisms extracellularly (64). P. gingivalis also antagonized the formation of NETs, specifically in the context of stimulation with the RPS mutants. While there are reports of NET induction by P. gingivalis (65, 66), the current study is consistent with that of Hirschfeld et al., who reported potent induction of NETs by S. gordonii but not P. gingivalis (67). Lactoferrin is another antimicrobial granule protein produced by neutrophils (44), and P. gingivalis inhibited lactoferrin degranulation in response to stimulation with the RPS mutants, indicating P. gingivalis can impact both secondary specific granules and azurophilic granules.

Effective neutrophil responses are important both to maintain immunity at densely populated gingival surfaces and for clearance of microbes that gain systemic access. P. gingivalis and S. gordonii are synergistically pathogenic both in murine models of periodontal disease (15, 22) and in abscesses (13, 31). In the current study, we found that P. gingivalis could enhance the survival of S. gordonii in a murine abscess model, although this synergy was not strictly dependent on P. gingivalis-mediated disruption of neutrophil functions. However, loss of RPS production rendered S. gordonii more susceptible to neutrophil killing, and streptococcal cell survival could be rescued by P. gingivalis. This effect was independent of gingipain activity and thus represents a novel mechanism of immune subversion by P. gingivalis. The finding that this synergistic mechanism is only manifested in the absence of RPS raises the question of biological relevance, as most sequenced strains of oral streptococci possess the RPS-encoding operon. However, an *in silico* interrogation revealed 4 of 13 fully sequenced S. gordonii strains lack homologs of SGO_2020 and SGO_2024 (Fig. S8), and moreover, these genes were identified in only 36 of 88 strains for which sequences are available as contigs. Strains lacking SGO_2020 and SGO_2024 can be predicted to have a competitive advantage in coinfections with P. gingivalis.

10.1128/mbio.00658-23.8 FIG S8 Gene cluster in the SGO_2020/2024 region drawn using the web server SyntTax (T.-H. Chang, H.-Y. Huang, J. B.-K. Hsu, S.-L. Weng, et al., BMC Bioinformatics 14:S4, 2013, https://doi.org/10.1186/1471-2105-14-4). The algorithm considers a score of 100 when the blastp result of a query amino acid sequence matches a known sequence exactly. The amino acid sequences of SGO_2020 (A) and SGO_2024 (B) were aligned against 13 fully sequenced strains of Streptococcus gordonii. Both these proteins returned a score of >95 with 9 strains; however, 4 strains returned a score of 13 with SGO_2020 and no score with SGO_2024. Download FIG S8, PDF file, 0.7 MB. Copyright © 2023 Pandey et al. 2023 Pandey et al. https://creativecommons.org/licenses/by/4.0/ This content is distributed under the terms of the Creative Commons Attribution 4.0 International license.

In summary, using Tn-Seq we have identified S. gordonii CoDE genes in communities with P. gingivalis, as well as an understudied class of genes that, when deleted, increase fitness in communities. A number of the latter genes were responsible for the production of RPS in S. gordonii. The presence of P. gingivalis compensated for the loss of RPS by impeding neutrophil mobilization, degranulation, and NET formation. Strains of S. gordonii that do not produce resource-intensive RPS may thus have increased fitness in communities with synergistic organisms.

## MATERIALS AND METHODS

### Construction of a genome-wide S. gordonii Tn*5* transposon library.

An Ez-Tn*5* transposon was constructed by mosaic end (ME)-tailed PCR using a modification of the methods for custom transposon construction (https://www.lucigen.com/docs/manuals/MA126E-EZ-Tn5-Transposase.pdf) for use with a Lucigen EZ-Tn*5*TM <KAN-2> insertion kit (LGC Biosearch Technologies). Erythromycin resistance encoded by *ermB* from pAMbeta1 (GenBank accession no. NC_013514.1) was used for the transposon marker. An ~1,040-bp fragment was amplified with primers TnErmF and TnErmR (see Table S2 in the supplemental material) and high-fidelity Platinum *Taq* (Thermo Fisher) using template pVA749 plasmid DNA (68), which carries the *ermB* gene with its promoter and putative ribosomal binding site. The constructed primers (Invitrogen) were phosphorylated at the 5′ end and carried a 19-bp Tn*5* ME recognition sequence followed by nucleotides designed to anneal to the erythromycin resistance determinant. The reaction product was designated the EZ-Tn*5* Erm transposon and was confirmed by DNA sequencing.

10.1128/mbio.00658-23.10 TABLE S2 Primers used in this study. Download Table S2, DOCX file, 0.02 MB. Copyright © 2023 Pandey et al. 2023 Pandey et al. https://creativecommons.org/licenses/by/4.0/ This content is distributed under the terms of the Creative Commons Attribution 4.0 International license.

The EZ-Tn*5* Erm transposon was used for *in vitro* random insertion into S. gordonii Challis CH1 genomic DNA using Lucigen EZ-Tn*5* transposase (LGC Biosearch Technologies). The *in vitro* insertion reaction mixtures contained 1 μg of target genomic S. gordonii DNA, 1× reaction buffer, 1 U EZ-Tn*5* transposase, and 1 μg of EZ-Tn*5* Erm transposon. The molar ratio of transposon to target DNA was ~2,000:1 per reaction. Insertion reaction mixtures were incubated for 2 h at 37°C and terminated by addition of 10× stop solution, followed by a 10-min incubation at 75°C according to the Lucigen EZ-Tn*5* insertion kit protocol. DNA was recovered, and nucleotides were added to the 3′ end of the inserted transposon DNA using T4 DNA polymerase (1 U), deoxynucleoside triphosphate (dNTP) mix (10 mM), 1× buffer 2.1 (New England Biolabs), and bovine serum albumin (BSA) (50 μg/mL). Polymerase reaction mixtures were incubated for 20 min at 12°C and terminated by incubation for 10 min at 75°C. Newly synthesized DNA ends were ligated by addition of T4 DNA ligase (1 U) and 1× T4 DNA ligase buffer (New England Biolabs) and incubated for 16 h overnight at 16°C. The resulting purified transposase-free genomic DNA carrying Tn*5*-*ermB* insertions throughout S. gordonii chromosomal DNA was used to transform competence-stimulating peptide (CSP)-induced competent S. gordonii cells.

S. gordonii was grown in Todd-Hewitt (TH) broth to an approximate optical density at 600 nm (OD_600_) of 0.1. DNA from the above ligation reactions and 1 μL of 1-mg/mL stock in double-distilled water (ddH_2_O) of CSP (Mimetopes) were added to 300 μL of S. gordonii cells. Transformation reaction mixtures were incubated aerobically in 5% CO_2_ at 36°C for 90 min. The entire transformation reaction was plated onto TH agar plates containing 5 μg/mL erythromycin and grown aerobically in 5% CO_2_ for 48 h. Colonies were recovered and suspended in 5 mL TH broth. Approximately 37,300 colonies were pooled from 3 independent transformations. Two hundred-microliter aliquots of the colony mixtures were added to equal volumes of glycerol and immediately frozen at −80°C.

For verification of the transposon inserts, 100 colonies were randomly chosen from the three starter libraries to determine the presence and chromosomal copy number of the integrated transposon. Genomic DNA preparations from each selected colony, as well as from the S. gordonii parent as a negative control, were digested with XbaI and analyzed by Southern hybridization using digoxigenin-labeled EZ-Tn*5* Erm as a probe. Each of the library colonies had a single reactive band, indicating a single transposon per colony; no bands were detected in the parental S. gordonii DNA.

### Construction of mutant strains.

Mutants with deletions in genes SGO_2020, SGO_2024, and SGO_1802 were constructed by allelic exchange as described previously (69). In brief, for the Δ2020 and Δ2024 strains, regions flanking the desired deletions were amplified (primers in Table S2) and cloned in E. coli DH5α in either plasmid pBluescript SK^+^ (for Δ2020) or pGem7 (for Δ2024) carrying a promoterless and terminatorless *aad9* gene (encoding spectinomycin resistance) or *aad9*, respectively. Cloned plasmids were verified by sequencing. The Δ1802 strain was constructed by commercial (GenScript) synthesis and cloning of an ~1,880-bp EcoRI-flanked fragment of the desired DNA, in which a 518-bp internal region of SGO_1802 was replaced with a promoterless and terminatorless *aad9* gene, into the *EcoRI* site of pUC57. The double-stranded DNA (dsDNA) inserts were released from plasmid vectors by restriction enzyme digestion and transformed into parental S. gordonii serum-competent cells. Putative transformants were selected with 250 μg/mL spectinomycin and confirmed by both Southern blot analysis and nucleotide sequencing. Loss of mRNA expression from target genes and expression from downstream genes was confirmed by RT-PCR.

### *In vivo* mouse abscess screen.

All murine experiments were approved by the University of Louisville Institutional Animal Care and Use Committee. Eight- to 10-week-old BALB/c mice were inoculated subcutaneously in the thigh with 0.1 mL of the S. gordonii library suspended in phosphate-buffered saline (PBS). Two groups, 10 mice each, were inoculated with either 2 × 10^9^ CFU of the library alone (Sg-only group) or 1 × 10^9^ CFU library plus 1 × 10^9^ CFU of P. gingivalis (Pg+Sg group). After 3 days, mice were euthanized, and abscesses were surgically excised and used to inoculate 2 mL of TH broth supplemented with 10 μg/mL erythromycin. Cultures were grown anaerobically until turbid, and 1.5 × 10^9^ bacteria were collected for DNA isolation.

### Construction of DNA sequencing libraries.

DNA isolation was performed with the Wizard genomic DNA isolation kit (Promega). A total of 5 μg of DNA suspended in 130 μL of Tris-EDTA (TE) was sheared by sonication (Covaris) to an average size of 300 bp and then purified and concentrated using a PCR cleanup kit (NEB Monarch) according to the manufacturer’s instructions. Poly(C) tails were added to sheared DNA using 1 μg DNA, 15 U TdT enzyme, dCTP (475 μM final concentration), and ddCTP (25 μM final concentration) in a total volume of 40 μL for 1 h at 37°C, followed by 75°C for 20 min. DNA was purified using the Monarch PCR cleanup kit according to the manufacturer’s instructions. DNA adjacent to each transposon insertion site was amplified by PCR using one primer targeting the poly(C) tail [poly(G) primer] (Table S2) and one targeting the end of the transposon (Tn end primer) (Table S2). For PCR, 2.5 U of AccuPrime Pfx DNA polymerase (Thermo Fisher), 1.5 μL of each primer (20 μM), and 250 ng template were used in a 50-μL reaction mixture. The cycling conditions were 95°C for 2 min, followed by 24 cycles of 95°C for 15 s, 55°C for 30 s, and 68°C for 2 min. A final 2-min extension at 68°C was also performed. PCRs were run on a 1.5% agarose gel, and a range from 250 to 1,000 bp was excised and purified using a gel extraction kit (NEB). Barcodes and primer sites for next-generation sequencing (Illumina) were added according to the manufacturer’s protocol.

### Sequencing and data analysis.

Single-end sequencing was performed on an Illumina Nextseq 500/550 75-cycle high-output kit v.2 (Illumina) at the University of Louisville Center for Genetics and Molecular Biology. Base calls were made using the BaseSpace FastQ v.1.0.0 application (Illumina). The quality of reads was assessed using FastQC (70). The majority of trailing G’s at the end of the reads were removed using a custom script, and the script was also used to remove the amplified transposon sequence (in full or partial form), comprising CATAACTTCTTTTACGTTTCCGCC. The trimmed reads were aligned to S. gordonii CH1 genome (accession no. NC_009785.1) using the STAR aligner (71) to generate BAM files. Raw insertion counts were generated by parsing the BAM files to determine the insertion locations for each read, and the total number of insertions for each of the bases in the genome was then computed. Using the number of insertions at each base in the genome, the total number of insertions in each gene was calculated by summing the number of insertions across all bases within the genomic coordinate boundaries of each gene. The total number of insertion counts per gene was input to the DESeq2 Bioconductor/R package (https://bioconductor.org/packages/release/bioc/html/DESeq2.html) using the recommended guidelines, including filtering out genes with total counts of less than 10 across all samples. Output included log_2_ fold change expression values and *P* values adjusted for multiple comparisons using the Benjamini-Hochberg procedure (72). This output was used as input for the generation of volcano plots using the EnhancedVolcano Bioconductor/R package (https://www.bioconductor.org/packages/release/bioc/html/EnhancedVolcano.html) and for functional enrichment analysis through the STRING Database v.11.5 (73) using a false-discovery rate (FDR) stringency of 25% and a minimum-interaction confidence score of 0.4 for network generation. For heat map generation, the raw count data were made homoscedastic using a regularized logarithm transformation as implemented by Love et al. (74). Gene count data for heat map generation were further converted into Z-scores and used as input into the ComplexHeatmap Bioconductor/R package (75). Visualization of sets of genes influencing fitness in common among experimental groups was performed using Venn diagrams generated through the ggvenn R package (76). COG figures were generated by uploading the coding sequences of S. gordonii genes into the eggNOG-mapper v.2.19 (77) to determine COG categories for each gene. Any genes that did not produce a COG category through eggNOG-mapper were manually parsed using EggNOG database v.5.0 (78). Genes that did not produce a COG ID, or which were identified as either category S (function unknown) or R (general function prediction only), were recategorized as “poorly characterized,” while genes falling under multiple categories were recategorized as “multiple.”

### Human neutrophil isolation and bacterial culture.

Blood was drawn from healthy donors, and neutrophils were purified using plasma-Percoll gradients as previously described (79) and in accordance with the guidelines approved by the Institutional Review Board of the University of Louisville. The purity of the isolated cell fraction was determined by cytospins, Giemsa staining, and microscopic evaluation to be ≥95%. Cell viability was confirmed by trypan blue exclusion and showed ≥97% viability. Neutrophils were routinely cultured in RPMI medium (Gibco) containing 10% heat-inactivated fetal bovine serum.

Streptococcus gordonii CH1 and isogenic mutant strains were grown in brain heart infusion (BHI) supplemented with yeast extract (1 mg/mL). Porphyromonas gingivalis ATCC 33277 was cultured in tryptic soy broth (TSB) supplemented with yeast extract (1 mg/mL), hemin (5 μg/mL) and menadione (1 μg/mL). Bacteria were cultured anaerobically to late log phase (OD_600_ of 0.8), harvested by centrifugation (5,000 × *g*), and washed once in prereduced PBS.

### Anthony’s staining.

Ten microliters of 1% crystal violet was spotted on a glass slide and mixed with a loopful of the bacterial culture. After smearing, the slide was dried and rinsed with 20% copper sulfate.

### Bacterial survival in abscesses.

BALB/c mice (8- to 10-week-old; 8 mice per group) were injected subcutaneously in the thigh with either 3 × 10^9^ S. gordonii cells (Sg-only group) or 1.5 × 10^9^ S. gordonii cells and 1.5 × 10^9^ P. gingivalis cells (Pg+Sg group) in a total volume of 100 μL. Abscesses were collected 24 h postinjection, and genomic DNA (gDNA) was isolated using the Wizard genomic DNA isolation kit. qRT-PCR was performed using S. gordonii or P. gingivalis 16S rRNA gene primers (Table S2), and bacterial numbers were calculated from a standard curve of gDNA isolated from known numbers of organisms.

### *In vivo* neutrophil mobilization assay.

BALB/c mice (8 to 10 weeks old; 8 mice per group) were injected subcutaneously in the thigh with either 1.5 × 10^9^ S. gordonii cells (Sg-only group) or 1.5 × 10^9^ S. gordonii cells and 1.5 × 10^9^ P. gingivalis cells (Pg+Sg group) in a total volume of 100 μL. After 4 h, each mouse was injected intraperitoneally with 150 μL (40 mg/mL) of IVISbrite MPO 425 chemiluminescent probe in RediJect solution (PerkinElmer). After 10 min of incubation and 300 s of exposure, *in vivo* imaging was performed using a Spectral Ami HT (Spectral Instruments Imaging). Image validation, acquisition, and data analysis were performed with Aura imaging software.

### Neutrophil survival assay.

The ability of bacteria to survive in the presence of neutrophils was assayed essentially as described previously (80). Bacteria were incubated with freshly isolated human neutrophils (1 × 10^6^ cells/mL) at 37°C for 20 min in 5% CO_2_. Extracellular, nonadherent bacteria were removed by washing, while extracellular adherent bacteria were killed by the addition of gentamicin (300 μg/mL), erythromycin (10 μg/mL), and metronidazole (200 μg/mL) for 1 h. Internalized bacteria were released by hypotonic lysis for 20 min. Serial dilutions of the lysates were plated on BHI or TS agar and cultured anaerobically for CFU enumeration.

### Chemiluminescent detection of ROS.

ROS production was measured by luminol-dependent chemiluminescence in a Spectramax L luminometer (Molecular Devices). Briefly, neutrophils were suspended in PBS containing 90 mM calcium chloride, 50 mM magnesium chloride, 750 mM glucose, 125 μM luminol, and 100 U/mL horseradish peroxidase in 96-well white plates (Costar). Prewarmed stimuli were added to the neutrophils, and ROS production (measured in relative light units [RLU]) was recorded at 37°C continuously for 1 h as total ROS. Intracellular ROS was detected with superoxide dismutase (SOD) (5 μL, 3 U/μL), while luminol chemiluminescence in the absence of SOD represents total ROS (extracellular and intracellular).

### NET formation.

A NETosis assay kit (Abcam), following the manufacturer’s instructions, was utilized. Briefly, freshly isolated human neutrophils (1 × 10^6^ cells/mL) were resuspended in prewarmed NET assay buffer, distributed in 24-well plates, and reacted with bacteria or phorbol myristate acetate (PMA) as a positive control for 4 h. Soluble, non-NET-associated elastase was removed by washing, and NETs were treated with S7 nuclease (15 U/mL) for 30 min at 37°C. After inactivation of nuclease with 500 nM EDTA and centrifugation to remove cell debris, neutrophil elastase substrate (1:1) was added and the mixture was incubated for 3 h at 37°C. Absorbance was recorded at 405 nm.

### Opsonization.

Bacterial cells were resuspended in Hanks balanced salt solution (HBSS) (without Ca^2+^/Mg^2+^) and opsonized with 10% freshly isolated human serum, pooled from 10 healthy donors, for 1 h at 37°C with gentle rocking. Complement- and antibody-mediated opsonization were determined by flow cytometry. Briefly, cells were stained with anti-C3b/iC3b conjugated with fluorescein isothiocyanate (FITC) (1:200), anti-IgG conjugated with phycoerythrin (PE) (1:100), or anti-IgM conjugated with allophycocyanin (APC) (1:50) (all from BioLegend) for 1 h at 4°C in the dark. Cells were washed once with PBS and fixed in 2% paraformaldehyde prior to flow cytometry. Data were acquired using a BD fluorescence-activated cell sorter (FACS) Celesta flow cytometer and analyzed using FlowJo software.

### Degranulation assays.

Degranulation was measured by the release of granule proteins by enzyme-linked immunosorbent assay (ELISA). Briefly, neutrophils (0.2 × 10^6^ per well) were suspended in RPMI 1640 containing 10% fetal bovine serum (FBS) in 96-well plates and stimulated with bacteria at 37°C for 4 h. Separately, neutrophils were also stimulated with latrunculin (1 μM) for 30 min, followed by fMLF (*N*-formylmethionyl-leucyl-phenylalanine) (1 μM) for 10 min, to allow for maximal degranulation. Elastase or lactoferrin release was measured in cell-free supernatants by ELISA (elastase/ELA2 DuoSet ELISA kit from R&D and lactoferrin [HLF2] human ELISA kit from Abcam) following the manufacturers’ instructions.

### Phagocytosis.

Human neutrophils (4 × 10^6^ cells/mL) were challenged with either opsonized or nonopsonized, CFSE-labeled S. gordonii in combination with unlabeled P. gingivalis. Cells were incubated in a shaking water bath at 37°C for 30 min and pelleted at 600 × *g* for 5 min, followed by rinsing with PBS and fixing with 1% paraformaldehyde. The neutrophil plasma membrane was stained for 10 min with wheat germ agglutinin (WGA) (Invitrogen), and cells were analyzed using an Amnis ImageStream imaging cytometer (Millipore). Data were analyzed using IDEAS image data exploration and analysis software v.6.0 (Amnis), and quantitation of fluorescence was done with ImageJ.

### Isolation and purification of streptococcal receptor polysaccharide.

S. gordonii cells were extracted overnight with 0.1% Triton X-100 in 0.9% NaCl, recovered by centrifugation (4,250 × *g*, 4°C, 25 min), and washed four times with PBS containing 0.02% sodium azide. Cells were resuspended in a mixture of 50 mM MgCl_2_·6H_2_O and 20 mM NaOAc·3H_2_O buffer (pH 7.3) and digested overnight with 7,500 U of proteinase K at 37°C. The cell pellet was recovered by centrifugation and further extracted for 24 h with 6 M guanidine–10 mM Tris-HCl (pH 8) buffer with vigorous shaking at room temperature. The extracted cells were suspended in 10 mM sodium phosphate buffer (pH 6.5) containing 10 mM MgCl_2_ and 0.02% sodium azide and digested overnight with 100 U of mutanolysin at 37°C (Sigma). The digest was placed on an ice bath, and cold trichloroacetic acid (TCA) was added to a final concentration of 5%. Cells were removed by centrifugation, and the pH of the supernatant was brought up to neutral with Tris base. The resulting salts and the buffer were removed by extensive 3-day dialysis (1,000-molecular-weight-cutoff [MWCO] dialysis bag) against several exchanges of H_2_O at 4°C. The dialyzed supernatants and the cell pellets were freeze-dried.

### Glycosyl composition analysis.

The glycosyl composition of the cell wall soluble polysaccharides was determined by preparing trimethylsilyl (TMS) methyl glycosides after 18 h of acidic methanolysis (1 M HCl-MeOH at 80°C) in the presence of the internal standard *myo*-inositol as previously described (81). Briefly, 400 μg of dry samples was converted to methyl glycosides, re-*N*-acetylated (methanol-pyridine-acetic anhydride at 2:1:1 [vol/vol/vol], 1 h, 100°C), and trimethylsilylated (200 μL Tri-Sil HTP reagent [Thermo], 80°C, 30 min). The TMS derivatives were dissolved in 300 μL of hexane and analyzed by gas chromatography mass spectrometry (GC-MS) on a Hewlett-Packard HP5890 gas chromatograph equipped with mass selective detector 5970 MSD, using an EC-1 fused silica capillary column (30 m by 0.25-mm inside diameter [i.d.]) and temperature cycle at 80°C for 2 min, followed by ramping to 160°C at 20°C/min, then to 200°C at 2°C/min, followed by an increase to 250°C at 10°C/min with an 11-min hold. The moles percent of the individual glycosyl residues were calculated based on the response factors of the original neutral and amino sugar standards to the internal standard of *myo*-inositol.

### Statistical analyses.

Statistical analyses of the sequence data are described above in the individual sections. Other experiments were performed with at least three biological replicates and two technical replicates and analyzed with GraphPad Prism 9.0.

### Data availability.

Tn-Seq data have been deposited in GEO under accession no. GSE219019.
