# Supplementary material for: Impact of Polymicrobial Infection on Fitness of Streptococcus gordonii In Vivo
Source: mBio. 2023 Apr 12;14(3):e00658-23. doi: 10.1128/mbio.00658-23 (PMC10294625; doi:10.1128/mbio.00658-23)
Supplement: FIG S1 [file mbio.00658-23-s0001.pdf]

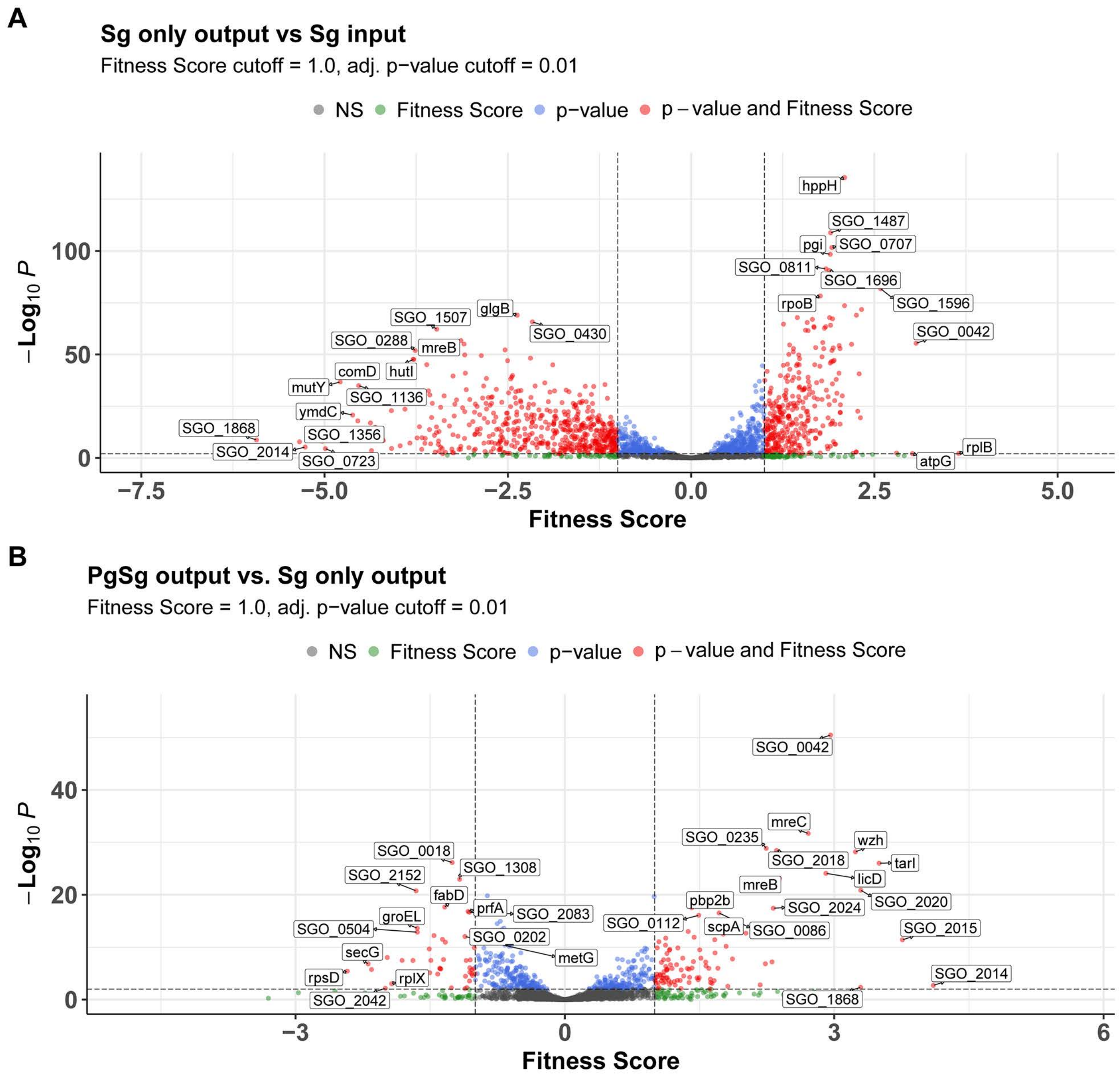

Figure S1. Volcano plots showing mean fitness scores ($\log_2$ difference in abundance in the co-infection group relative to the monoinfection group) and p-values (-$\log_{10}$) for *S. gordonii* (Sg) gene mutations in monoinfection (A) or co-infection with *P. gingivalis* (Pg) (B).
