# Supplementary material for: Impact of Polymicrobial Infection on Fitness of Streptococcus gordonii In Vivo
Source: mBio. 2023 Apr 12;14(3):e00658-23. doi: 10.1128/mbio.00658-23 (PMC10294625; doi:10.1128/mbio.00658-23)
Supplement: FIG S2 [file mbio.00658-23-s0002.pdf]

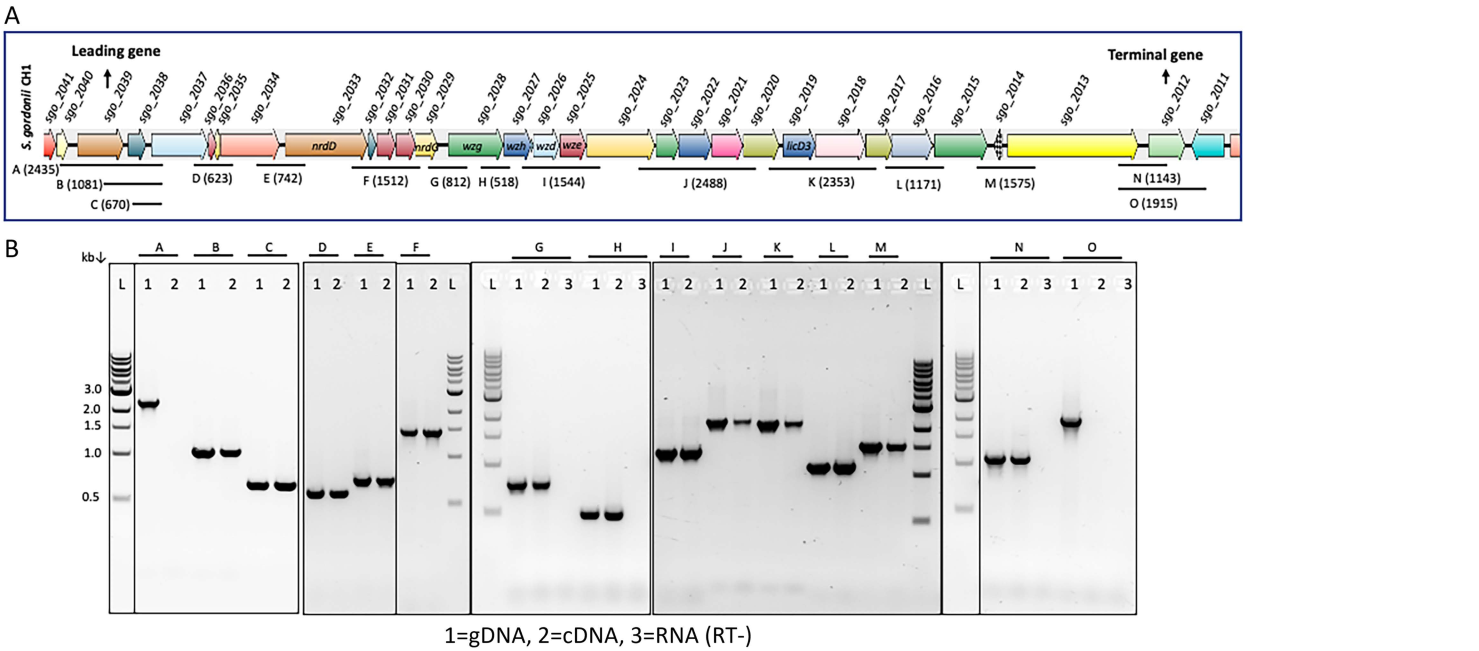

Figure S2. The RPS operon. PCR was used to investigate the operon arrangement of the RPS encoding genes in S. *gordonii*. Cells were disrupted using Trizol and a FastPrep-24 5G (MP Biomedicals). RNA was isolated using an RNeasy kit (Qiagen) and genomic (g) DNA contamination removed with a TURBO DNA-free kit (Invitrogen). A total of 2 µg RNA was converted to cDNA using a High-capacity cDNA synthesis kit (Applied Biosystems). Primers (A-O) are listed in Table S3, and positions along with predicted product size are indicated in the gene cluster diagram (A). RT-PCR, PCR with gDNA and a control with no RT are shown by agarose gel electrophoresis in (B). The arrows in (A) indicate the leading gene (SGO_2039) and terminal gene (SGO_2012) of the operon.
