# Supplementary material for: Impact of Polymicrobial Infection on Fitness of Streptococcus gordonii In Vivo
Source: mBio. 2023 Apr 12;14(3):e00658-23. doi: 10.1128/mbio.00658-23 (PMC10294625; doi:10.1128/mbio.00658-23)
Supplement: FIG S3 [file mbio.00658-23-s0003.pdf]

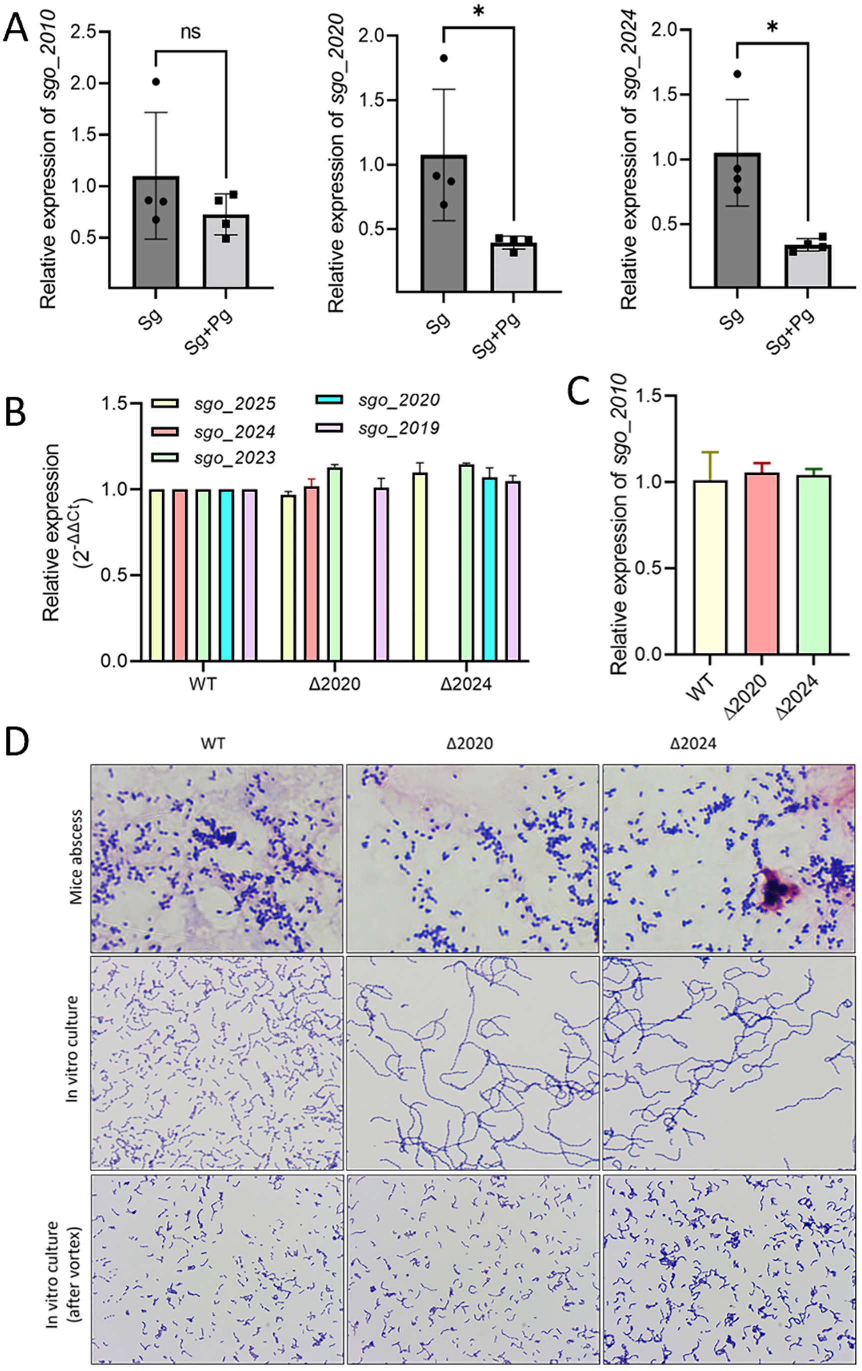

Figure S3. A) qRT-PCR of SGO_2010 (outside the RPS operon), SGO-2020, and SGO_2024 mRNA expression in *S. gordonii* isolated from either *S. gordonii* alone or *S. gordonii* + *P. gingivalis* abscess material. cDNA was prepared as described in the Figure S2 legend. mRNA levels were normalized to 16S RNA and expressed relative to *S. gordonii* alone. Data are means +/- SD from 4 biological replicates. *$p < 0.05$ by Mann Whitney U test. B) Confirmation of non-polar mutations in Δ2020 and Δ2024. mRNA extraction and cDNA synthesis were performed as described in Figure S2 legend. qRT-PCR was performed for the genes downstream of the deleted genes within the RPS operon (B), and a gene (SGO_2010) downstream of the RPS operon (C). Values are expression levels relative to parental *S. gordonii* CH1 (WT). Primers are in Table S2. D) Gram staining of *S. gordonii* CH1 (WT), Δ2020 and Δ2024 following recovery from mouse abscesses, in vitro culture or in vitro culture following vortexing for 10 s.
