# Supplementary material for: Impact of Polymicrobial Infection on Fitness of Streptococcus gordonii In Vivo
Source: mBio. 2023 Apr 12;14(3):e00658-23. doi: 10.1128/mbio.00658-23 (PMC10294625; doi:10.1128/mbio.00658-23)
Supplement: FIG S4 [file mbio.00658-23-s0004.pdf]

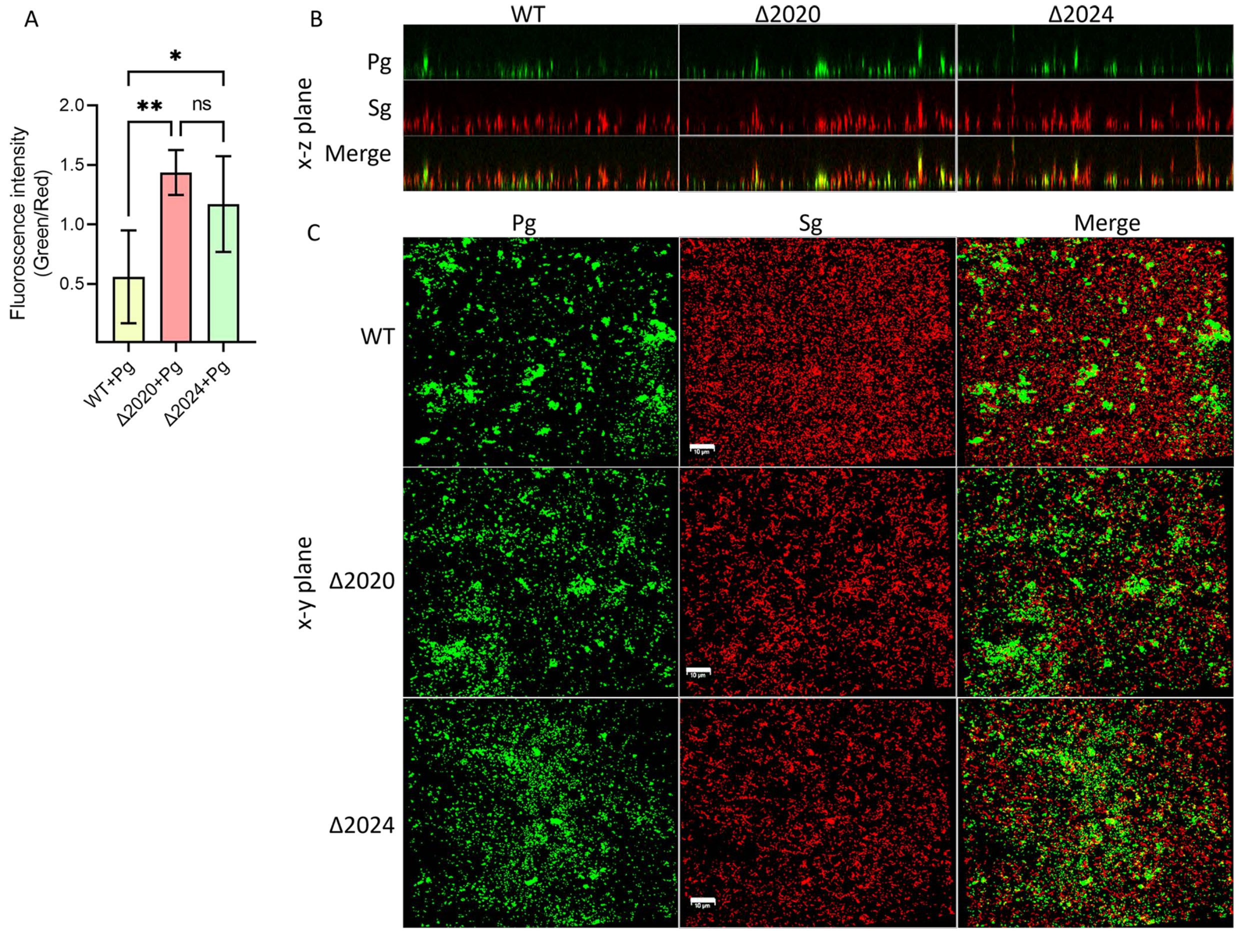

Figure S4. Community formation of *P. gingivalis* with *S. gordonii*. *P. gingivalis* (CFSE-labelled, green) was reacted with a substratum of *S. gordoni*i WT and Δ2020 and Δ2024 mutants (hexidium iodide-labelled, red) for 18 h and imaged by confocal microscopy. A) Ratio of P. *gingivalis*-*S. gordonii* biovolume from 6 random fields. Data are means +/- SD. * p<0.05, ** p<0.01 using ANOVA with Tukey's multiple comparisons test. B) and C) x-z and x-y projections of reconstructed images using Volocity software. Bar represents 10 µm.
