# Supplementary material for: Impact of Polymicrobial Infection on Fitness of Streptococcus gordonii In Vivo
Source: mBio. 2023 Apr 12;14(3):e00658-23. doi: 10.1128/mbio.00658-23 (PMC10294625; doi:10.1128/mbio.00658-23)
Supplement: FIG S5 [file mbio.00658-23-s0005.pdf]

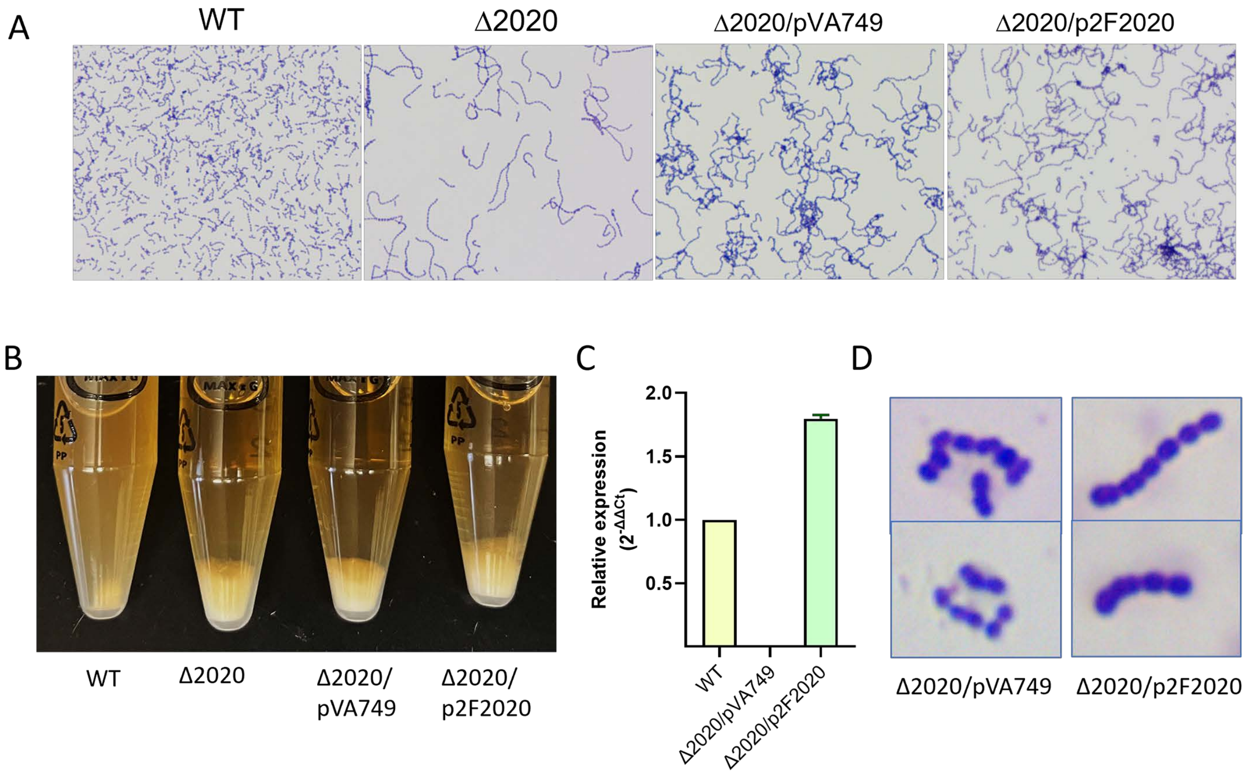

Figure S5. Complementation of Δ2020 with SGO_2020 in trans. Strains are: *S. gordonii* CH1 (WT); Δ2020 mutant; Δ2020 complemented in trans with plasmid pVA749 containing SGO_2020, along with the *S. mutans ldh* promoter upstream of the native putative ribosomal binding site and ATG start; and Δ2020 containing empty pVA749 vector. A) Gram-staining, B) growth in BHI medium, C) qRT-PCR for SGO_2020 mRNA (primers in Table S2), and D) RPS production visualized by Anthony's stain. Although the complemented mutant produced SGO_2020 mRNA, phenotypic complementation was not achieved.
