# Supplementary material for: Impact of Polymicrobial Infection on Fitness of Streptococcus gordonii In Vivo
Source: mBio. 2023 Apr 12;14(3):e00658-23. doi: 10.1128/mbio.00658-23 (PMC10294625; doi:10.1128/mbio.00658-23)
Supplement: FIG S6 [file mbio.00658-23-s0006.pdf]

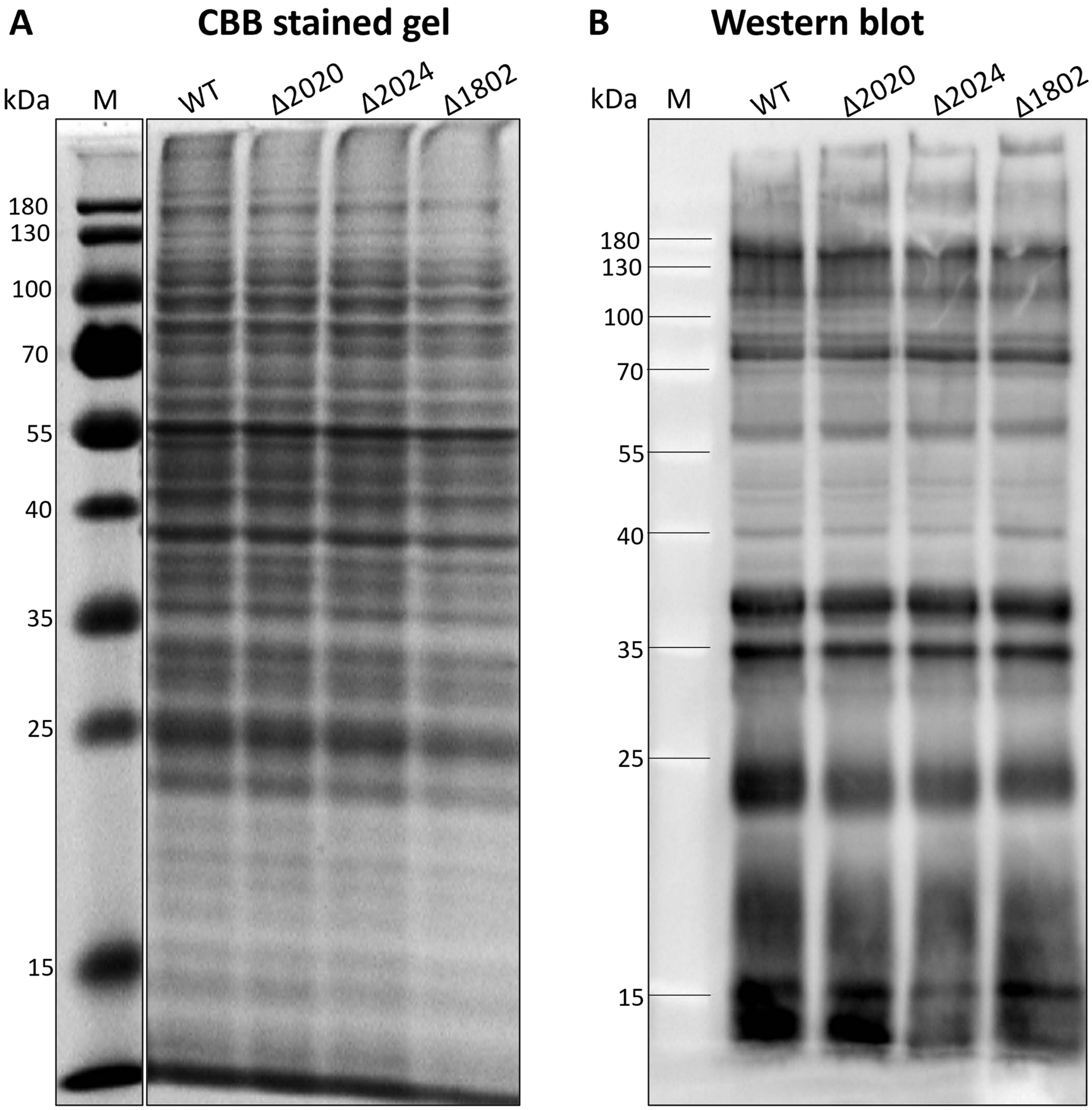

Figure S6. Protein expression and antigenic profile in *S. gordonii* strains. Bacteria were grown in BHI broth to OD600 ~0.8. Equal amounts of cells were harvested, washed and resuspended 50 mM Tris-Cl pH 7.6, 150 mM NaCl, and 5 % glycerol. Cells were mechanically disrupted using a FastPrep-24 5G. The lysate was centrifuged at 10,000 rpm for 5 min, the pellet dissolved in SDS-PAGE sample, separated by SDS-PAGE on a 12 % gel, and stained with Coomassie brilliant blue (A). For western blotting (B), proteins were electrotransferred (40 V, 2h) from unstained gels onto PVDF membranes. The membrane was blocked with 4% skimmed milk for 1 h, and washed x3 in TBST (TBS with 0.05% Tween 20) for 10 min. Primary rabbit antibody raised to *S. gordonii* strain G9B at 1:5000 was reacted o/n at 4°C, followed by washing again with TBST. Secondary antibody was anti-rabbit IgG-HRP (1:2000) and was reacted for 1 h at 4°C. The membrane was washed and developed using chemiluminescent substrate (SuperSignal West PicoPlus, Thermo Scientific). Images are representative of 3 biological replicates.
