# Supplementary material for: Impact of Polymicrobial Infection on Fitness of Streptococcus gordonii In Vivo
Source: mBio. 2023 Apr 12;14(3):e00658-23. doi: 10.1128/mbio.00658-23 (PMC10294625; doi:10.1128/mbio.00658-23)
Supplement: FIG S7 [file mbio.00658-23-s0007.pdf]

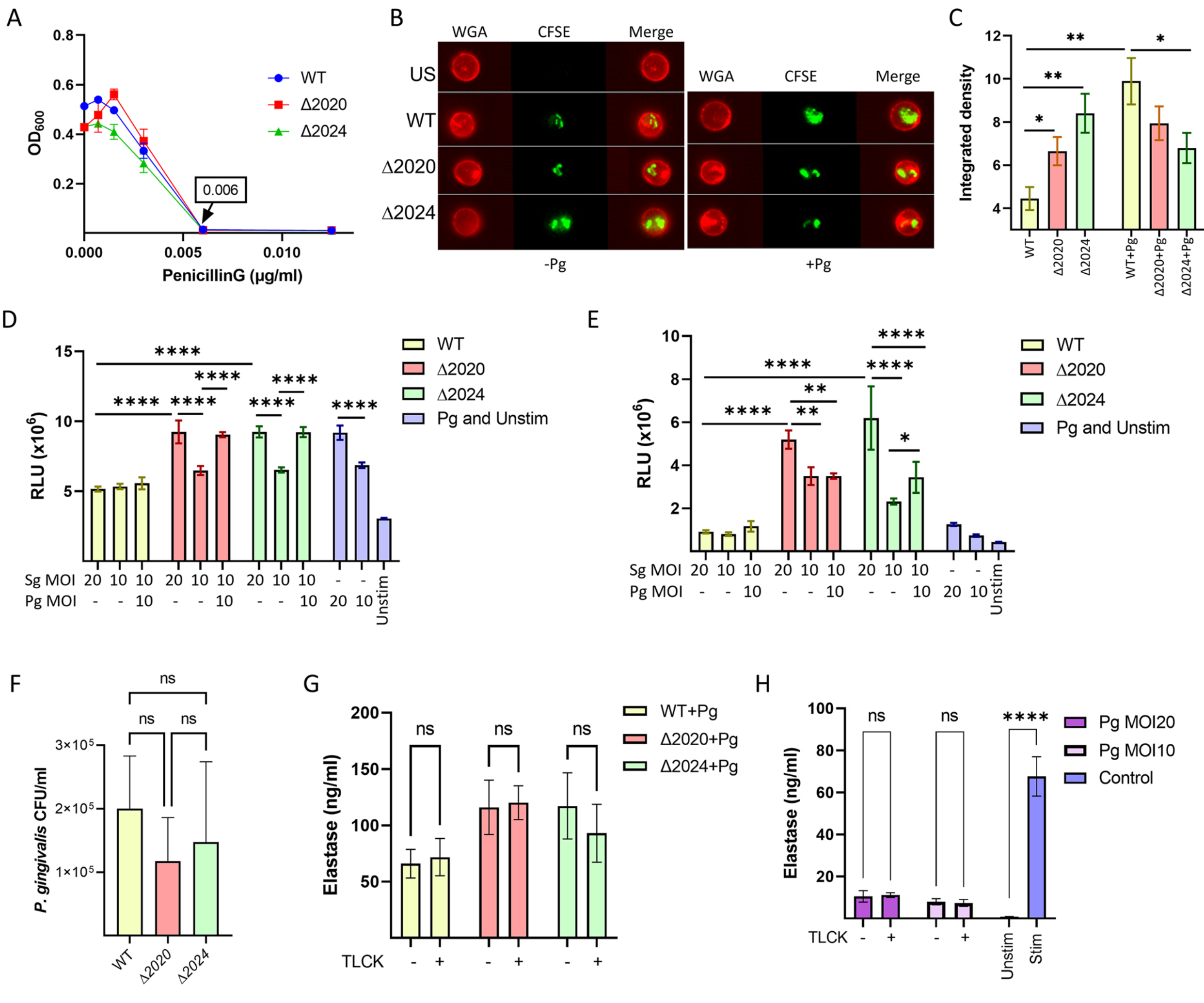

Figure S7. A) Penicillin sensitivity of *S. gordonii* strains. The minimum inhibitory concentration (MIC) was determined for penicillin (Sigma) using the broth microdilution method. are means +/- SD. B) Representative Amnis Imagestream images of WGA-stained human neutrophils (red) reacted for 30 min with CFSE-stained *S. gordonii* strains (green) with or without *P. gingivalis* as indicated. US indicates unstimulated cells. C) Plot of CFSE fluorescence associated with WGA-stained neutrophils (shown in B) from 60 cells. D) Induction of reactive oxygen species (ROS). Human neutrophils were challenged with *S. gordonii* strains with or without *P. gingivalis* at the MOIs indicated at 37°C, or were left unstimulated (Unstim). Total (D) and intracellular (E) ROS measured by lucigenin-elicited chemiluminescence. Intracellular ROS was detected in the presence of superoxide dismutase (SOD) to remove extracellular ROS. Summed integrated responses measured as relative light units/s (RLU) recorded over 1 h are shown. F) Viability of *P. gingivalis* from human neutrophils after co-infection with *S. gordonii*. Neutrophils were challenged with *P. gingivalis* and *S. gordonii* strains for 20 min in 5% CO2 each at MOI 10. Colony forming units (CFU) were calculated from post-lysis solutions incubated on TSB agar plates anaerobically.. G) Degranulation of elastase from human neutrophils. *P. gingivalis* cells were either pretreated with 100 µM of TLCK for 2 h at 37 °C anaerobically, or left untreated. H) elastase release after infection with *P. gingivalis* alone at the MOIs indicated for 4 h. Unstim is unstimulated. Stim is stimulated with latrunculin (1 µM) for 30 min and fMLF (1 µM), for 10 min. Data are means +/- SD. * p<0.05, ** p<0.01,*** p<0.05, **** p<0.001 using 2-way ANOVA with Tukey's multiple comparisons test. ns is not significant.
