# Supplementary material for: Impact of Polymicrobial Infection on Fitness of Streptococcus gordonii In Vivo
Source: mBio. 2023 Apr 12;14(3):e00658-23. doi: 10.1128/mbio.00658-23 (PMC10294625; doi:10.1128/mbio.00658-23)
Supplement: FIG S8 [file mbio.00658-23-s0008.pdf]

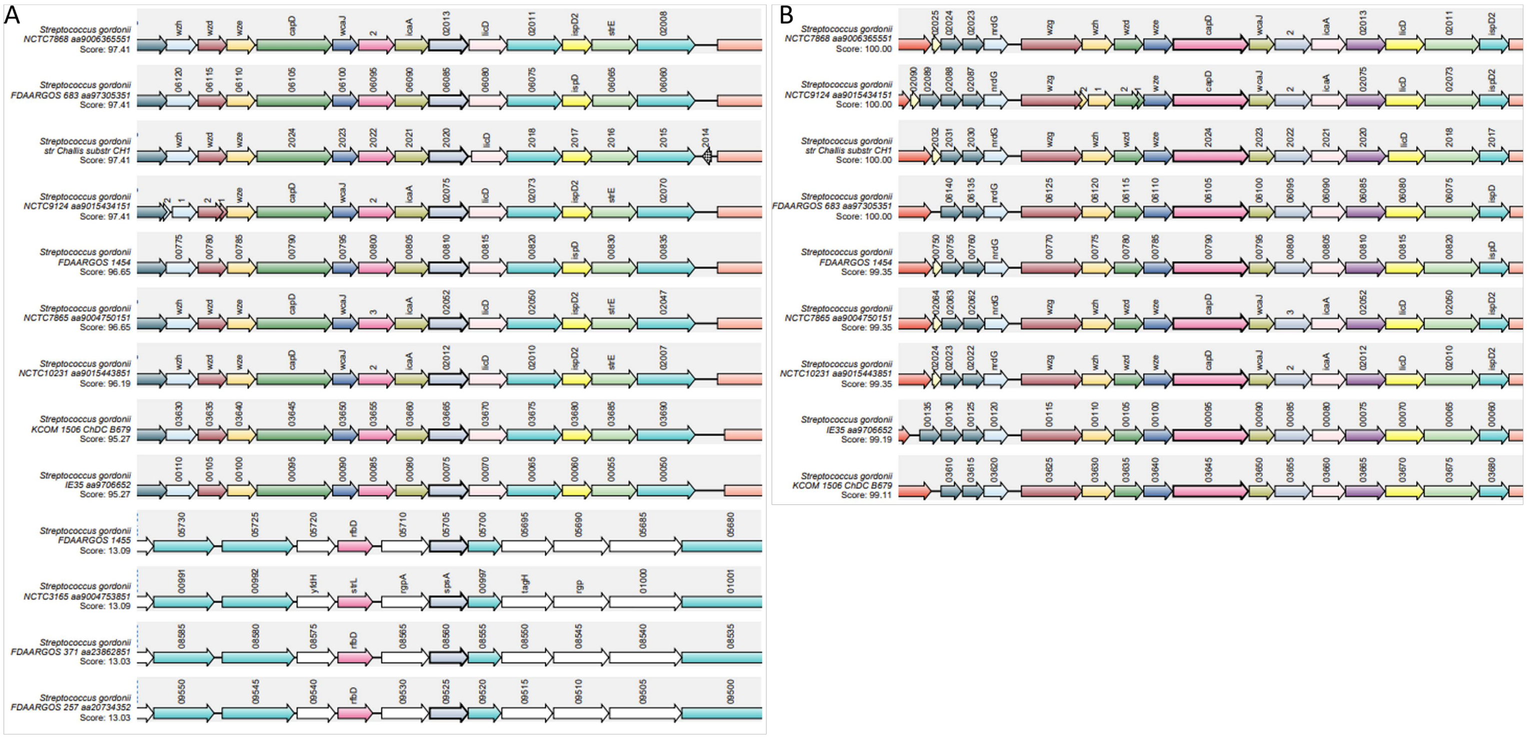

Figure S8. Gene cluster in the SGO_2020/2024 region drawn using the web server SyntTax (DOI: 10.1186/1471-2105-14-4). The algorithm considers a score of 100 when the BLASTP result of a query amino acid sequence matches a known sequence exactly. The amino acid sequences of SGO_2020 (Panel A) and SGO_2024 (Panel B) were aligned against 13 fully sequenced strains of *Streptococcus gordonii*. Both these proteins returned a > 95 score with 9 strains; however, 4 strains returned a score of 13 with SGO_2020, and no score with SGO_2024.
