# Supplementary material for: Impact of Polymicrobial Infection on Fitness of Streptococcus gordonii In Vivo
Source: mBio. 2023 Apr 12;14(3):e00658-23. doi: 10.1128/mbio.00658-23 (PMC10294625; doi:10.1128/mbio.00658-23)
Supplement: TABLE S2 [file mbio.00658-23-s0010.docx]

Table S2. Primers used in this study

| **Primer name** | **Sequence 5'- 3'** | **Purpose** |
| --- | --- | --- |
| SGO_1706_(16s)_FP1 | TATGACCTGGGCTACACACG | 16s rDNA; qRT-PCR |
| SGO_1706_(16s)_RP1 | GAGTTGCAGCCTACAATCCG |  |
| SGO_2019_qRT_F1 | TGCAGGCAGCCTATTTGGAA | Polar test; qRT PCR |
| SGO_2019_qRT_R1 | TATAGCCACCGTGTCGCAAA |  |
| SGO_2020_qRT_F1 | TATCTGGTGGGAGTCAGGCT | Polar and complementation test; qRT PCR |
| SGO_2020_qRT_R1 | ACACTTCCTGTCTAAAAGCCGT |  |
| SGO_2023_qRT_F1 | GGCGGGATGAGTTGCCTATT | Polar and complementation test; qRT PCR |
| SGO_2023_qRT_R1 | CACCCTCGACAACACCTTCA |  |
| SGO_2024_qRT_F1 | ATTGCAAAAGGGGGTCCTGT | Polar test; qRT PCR |
| SGO_2024_qRT_R1 | CCCCAGCTTGGATGACCAAT |  |
| SGO_2025_qRT_F1 | TAGAGCAGACAGGGACTCCG | Polar test; qRT PCR |
| SGO_2025_qRT_R1 | TCCCGTAAGCACCATAATTCCC |  |
| SGO_2010_qRT_F1 | CTTCTCAAACGGGACGGGAA | Polar test; qRT PCR |
| SGO_2010_qRET_R1 | TGACCCACTGGTCTCCAGAT |  |
| Op_2040-37-F | GGTGACCTTCAGCCTATTCCA | Operon confirmation; PCR product A, B and C |
| Op_2039-37-F | CAAGAGGAACTACCTCAGGCAA |  |
| Op_2038-37-F | AAACTCAGCCCTCTCAAGCC |  |
| Op_2037-R | CGTACCAACTGTTAATAAGGTGACT |  |
| Op_2037-34-F | ACAGAGGTTCATGACGGTGTC | PCR Product D |
| Op_2034-R | AATAAGGGATTGGCCACGCT |  |
| Op_2034-33-F | CGGGCTGTGGTTAGCAAGTA | PCR Product E |
| Op_2033-R | AAAGTCACGCTGGGTTCGAT |  |
| Op_2033-29-F | GTTGACGTGGTCAAACGGAC | PCR Product F |
| Op_2029-R | TTACGGACACCTTCGCCATC |  |
| Op_2029-28_F | TCGGGAAGGCCTCCTCTAAT | PCR Product G |
| Op_2029-28_R | CTCGCTGAGTCCACTGTCAA |  |
| Op_2028-27_F | CGGCAAGCCTGGATCAAATG | PCR Product H |
| Op_2028-27_R | CAAGTCGCGCACCTTCTTTT |  |
| Op_2027-26_F | AGAACCTCATGCAGAGCAGT | PCR Product I |
| Op_2025-24_R; | AGCGTTTTTGTGCCCTAGTT |  |
| Op_2024-23_F; | TACATGCTCCAGCCAAGAAGG | PCR Product J |
| Op_2021-20_R; | GTTGCTCGATAAACCTTCTTCCA |  |
| Op_2020-19_F; | GTCAGCGATGTGGTCTATGCT | PCR Product K |
| Op_2018-17_R | CCACCCGCAAATATCAACGC |  |
| Op_2017-16_F | AAGCGCGTGAAAATGCTCAA | PCR Product L |
| Op_2016-15_R | CAGCAAATTGGACAAGACGACA |  |
| Op_2015-14_F | TGCGAGCTCGTTGATGCTTA | PCR Product M |
| SGO_2013_qRT_R1 | ACCTGGCAACAATTCAGCCT |  |
| SGO_2013_qRT_F2 | AATCCCACGCCAAGTGACAG | PCR Product N |
| Op_2013-12_R | GGAGTAGCAGCTGGACCAAT |  |
| Op_2013-11_F(Rev) | ATCCGTGTCCACCTATCCCA | PCR Product O |
| Op_2011_R | CGTGAAGCATGAGACGACCT |  |
| PolyG | GTCTCGTGGGCTCGGAGATGTGTATAAGAGACAGGGGGGGGGGGGGGGGG | Library construction |
| Tn-Seq End | TCGTCGGCAGCGTCAGATGTGTATAAGAGACAGCATAACTTCTTTTACGTTTCCGCC | Library construction |
| TnErmF | CTGTCTCTTATACACATCTATCGAAACAGCAAAGAATGGCGG | Tn marker construction |
| TnErmR | CTGTCTCTTATACACATCTTGGAAGC GTCAGT AGTATACC | Tn marker construction |
| Xho2020up | ATCTCGAGAAATAGATATCTTAGGAAGCGCG | Deletion of SGO_2020 |
| Hin2020stopR | ATAAGCTTAAACCTTCTTCCATTATAAGTG GC |  |
| Bam2020DownF | ATGGATCCTCTAGTCAGCGATGTGGTCTATG |  |
| Sst2020DownR | TTGAGCTCCTTTGATTCTTTAGACAT |  |
| Xho2024up | ATCTCGAGGACAAATATTCAGTTAAGTGGAG | Deletion of SGO_2024 |
| Hin2024stopR | ATAAGCTTAAGTATCCACGATCATTAATATTAAGC |  |
| Bam2024DownF | ATGGATCCGAATGAGACTGAGTTGAAGCAAAG |  |
| Sst2024DownR | ATGAGCTCGACAACACCTTCACTCTTAAC |  |
| Bcl12020.2F | ATTGATCAGAATATTAAGGAAACAAAAATGATTAG | Complementation of Δ2020 |
| Bcl12020.3F | ATTGATCATGTCTATATGCCTCTGGGGTTG |  |
| Sst12020R | ATGAGCTCATTCTATTTTGCTTGTACTTCGC |  |
| PGN_16S_RT_FP | AGGAACTCCGATTGCGAAGG | 16s rDNA; qRT-PCR |
| PGN_16S_RT_RP | TCGTTTACTGCGTGGACTACC |  |
